# Dictionary of immune responses to cytokines at single-cell resolution

**DOI:** 10.1038/s41586-023-06816-9

**Published:** 2023-12-06

**Authors:** Ang Cui, Teddy Huang, Shuqiang Li, Aileen Ma, Jorge L. Pérez, Chris Sander, Derin B. Keskin, Catherine J. Wu, Ernest Fraenkel, Nir Hacohen

**Affiliations:** 1 Harvard–MIT Division of Health Sciences and Technology, Massachusetts Institute of Technology, Cambridge, MA USA; 2 Broad Institute of MIT and Harvard, Cambridge, MA USA; 3 Translational Immunogenomics Lab, Dana–Farber Cancer Institute, Boston, MA USA; 4 Department of Electrical Engineering and Computer Science, Massachusetts Institute of Technology, Cambridge, MA USA; 5 Department of Cell Biology, Harvard Medical School, Boston, MA USA; 6 cBio Center, Department of Data Science, Dana–Farber Cancer Institute, Boston, MA USA; 7 Department of Medical Oncology, Dana–Farber Cancer Institute, Boston, MA USA; 8 Department of Medicine, Brigham and Women’s Hospital, Harvard Medical School, Boston, MA USA; 9 Department of Biological Engineering, Massachusetts Institute of Technology, Cambridge, MA USA; 10 Department of Medicine, Massachusetts General Hospital, Harvard Medical School, Boston, MA USA; 11 Krantz Family Center for Cancer Research, Massachusetts General Hospital, Boston, MA USA; 12 Present Address: Faculty of Medicine, Harvard University, Boston, MA USA

**Keywords:** Computational biology and bioinformatics, Immunology, Systems biology

## Abstract

Cytokines mediate cell–cell communication in the immune system and represent important therapeutic targets^1–3^. A myriad of studies have highlighted their central role in immune function^4–13^, yet we lack a global view of the cellular responses of each immune cell type to each cytokine. To address this gap, we created the Immune Dictionary, a compendium of single-cell transcriptomic profiles of more than 17 immune cell types in response to each of 86 cytokines (>1,400 cytokine–cell type combinations) in mouse lymph nodes in vivo. A cytokine-centric view of the dictionary revealed that most cytokines induce highly cell-type-specific responses. For example, the inflammatory cytokine interleukin-1β induces distinct gene programmes in almost every cell type. A cell-type-centric view of the dictionary identified more than 66 cytokine-driven cellular polarization states across immune cell types, including previously uncharacterized states such as an interleukin-18-induced polyfunctional natural killer cell state. Based on this dictionary, we developed companion software, Immune Response Enrichment Analysis, for assessing cytokine activities and immune cell polarization from gene expression data, and applied it to reveal cytokine networks in tumours following immune checkpoint blockade therapy. Our dictionary generates new hypotheses for cytokine functions, illuminates pleiotropic effects of cytokines, expands our knowledge of activation states of each immune cell type, and provides a framework to deduce the roles of specific cytokines and cell–cell communication networks in any immune response.

## Abstract (web summary)

An extensive global transcriptomics analysis of in vivo responses to 86 cytokines across more than 17 immune cell types reveals enormous complexity of cellular responses to cytokines, providing the basis of the Immune Dictionary and its companion software Immune Response Enrichment Analysis.

## Main

Cytokines are a broad class of small, secreted proteins that act locally or systemically by binding to cognate receptors on target cells, which in turn trigger downstream signalling and orchestrate activities among cell types of the immune system. Cytokine-based therapies and cytokine antagonists are used to treat a wide range of disorders, including cancer and autoimmunity^14^. However, the large number of immune cell types and cytokines and complex cellular responses have made it challenging to elucidate in vivo immune responses to cytokines.

**Fig. 1:**
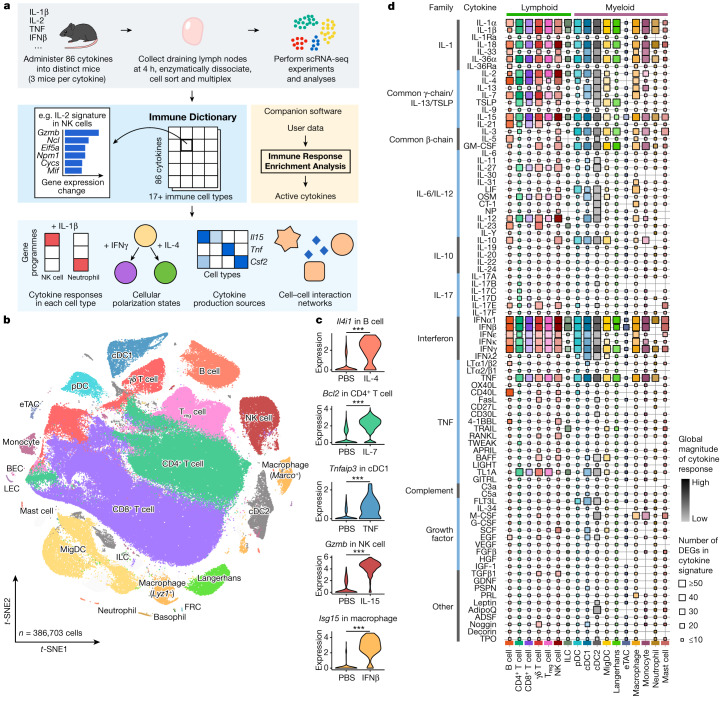
Generation of a scRNA-seq dictionary of gene expression signatures in more than 17 immune cell types in response to each of 86 cytokines in vivo. **a**, Schematic of the experimental and computational workflow. First row, data generation procedures; second row, illustration of the Immune Dictionary and its companion software IREA; third row, analyses of the Immune Dictionary. **b**, *t*-distributed stochastic neighbour embedding (*t*-SNE) map of all cells collected from lymph nodes after cytokine stimulation or without stimulation (PBS controls) coloured by cell-type identity. Cells were sorted to rebalance frequencies of major cell types. **c**, Violin plots of expression levels of well-established cytokine-responsive genes following PBS or cytokine treatment. ***False discovery rate (FDR)-adjusted *P* < 0.001, two-sided Wilcoxon rank-sum test. **d**, Quantitative representation of overall transcriptomic response levels in each cell type 4 h after cytokine stimulation compared with PBS controls. Each cell type is analysed independently and is represented by a distinct colour, following the colour codes in **b** and **c**. Colour saturation indicates the magnitude of the response. Size indicates the number of genes with significant differential expression (absolute log_2_(fold change (FC)) > 0.25 and FDR-adjusted *P* < 0.05, two-sided Wilcoxon rank-sum test) in each cytokine signature. Source data

## The Immune Dictionary

To obtain a comprehensive view of cellular responses to cytokines, we systematically profiled single-cell transcriptomic (single-cell RNA sequencing (scRNA-seq)) responses to 86 cytokines across more than 17 immune cell types in mouse lymph nodes in vivo to generate a large-scale perturbational scRNA-seq dataset of the immune system (Fig. 1a). The 86 cytokines represent most members of major cytokine families, including interleukin-1 (IL-1), common γ-chain/IL-13/thymic stromal lymphopoietin (TSLP), common β-chain, IL-6/IL-12, IL-10, IL-17, interferon (types I, II and III), tumour necrosis factor (TNF), complement and growth factor families, as well as representative molecules from other families with cytokine functions (for example, certain hormones) (Methods and Supplementary Table 1).

**Extended Data Fig. 1:**
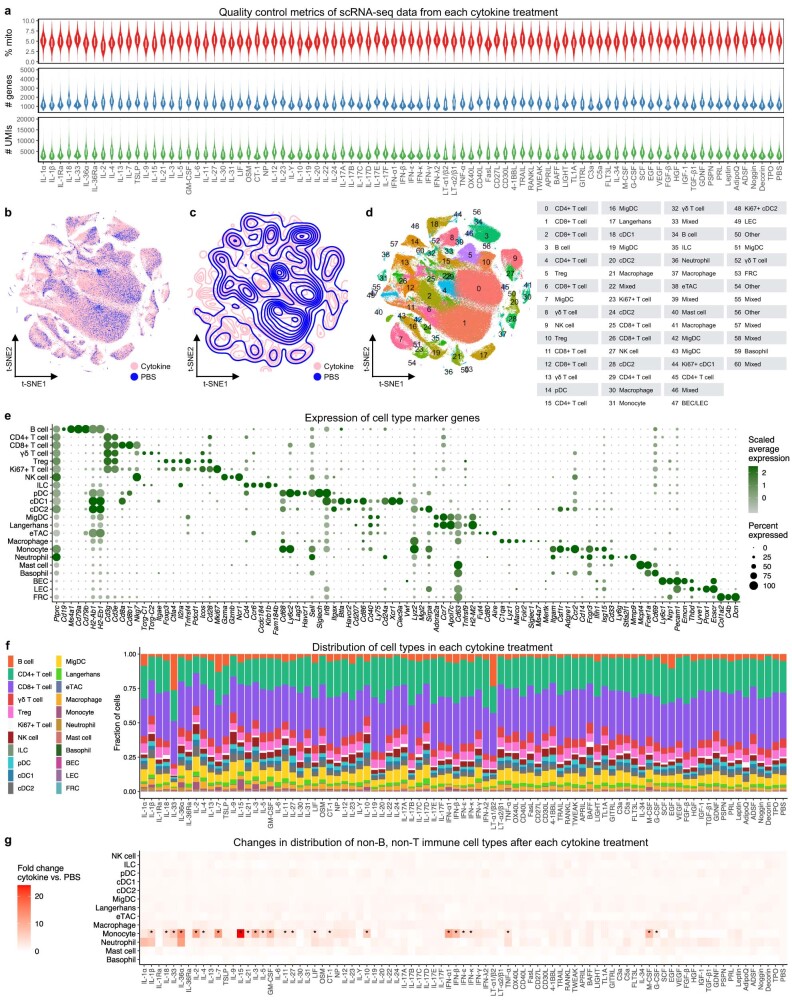
scRNA-seq data summary and quality metrics. **a**, Violin plots showing the distributions of percentage of mitochondrial gene content (top), number of genes detected (middle), and number of unique molecular identifiers (UMIs) detected (bottom) per cell post-quality control across cytokine or PBS treatment conditions. The interquartile range is shown as a white box inside each violin plot. n = 386,703 independent cells over 272 independent mice (3 mice per cytokine and 14 mice for PBS control). **b**, Two-dimensional t-SNE visualization of all cells (following the coordinates in Fig. 1b), colored by any cytokine treatment (pink) or PBS control (blue). **c**, Contour plot of the t-SNE map in **b**. **d**, t-SNE visualization of all cells, colored by level-1 Louvain clusters identified from global clustering. The dominant cell type associated with each cluster is indicated in the accompanying table. Each cluster is further divided into level-2 clusters to refine cell type identification (Supplementary Table 2). **e**, Dot plot showing the scaled average expression of cell type marker genes and percentage of cells expressing the genes in each annotated cell type. **f**, Cell type composition in each treatment. **g**, Changes in the fraction of non-B, non-T immune cell types after cytokine treatment relative to PBS controls. * denotes *P*-value < 0.05, one-sided Wilcoxon rank-sum test with FDR adjustment. Only CD3– and CD19– immune cells are shown as these cell types are not influenced by the cell sorting strategy used. Source data

**Extended Data Fig. 2:**
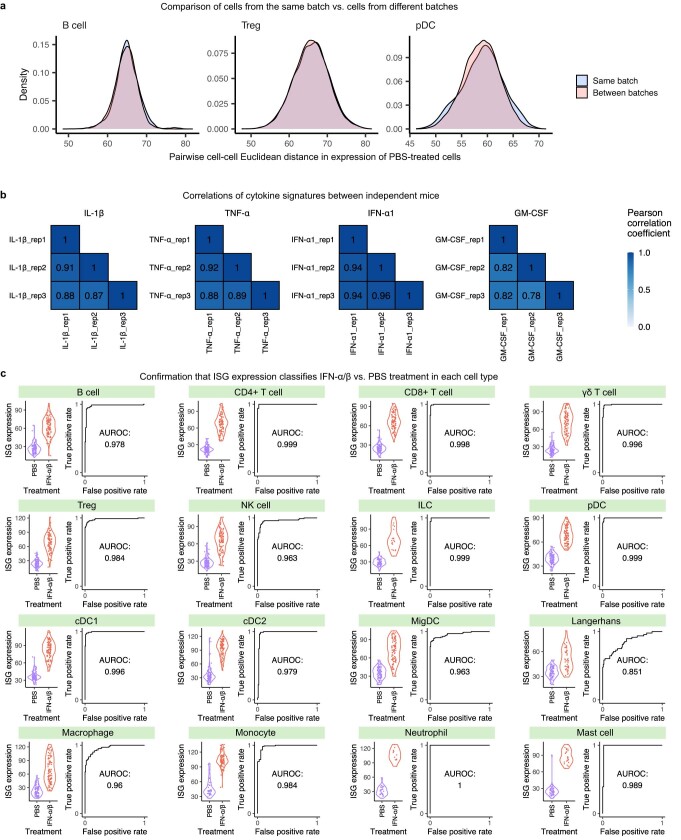
Additional scRNA-seq data quality metrics. **a**, Distributions of Euclidean distances between individual PBS-treated cells based on their transcriptomic profiles, colored by whether cells compared are from the same sample processing batch or across different sample processing batches. **b**, Pearson correlation coefficients between cytokine-induced gene expression signatures obtained from different animal replicates, using cDC1 as a representative example. **c**, As a positive control for the ability of each lymph node cell type to access the injected cytokines, IFN-α/β responses in each cell type are shown. At the single-cell level, ISG scores can accurately classify IFN-α/β-treated cells vs. PBS-treated cells based on areas under receiver operating characteristics (AUROC) curves in each cell type, confirming that the vast majority of the cells can access the injected cytokines. ISG expression in each cell is shown as individual dots and violin plots on the left side, and the classification accuracy is shown as ROC curves on the right side. Source data

We injected each freshly reconstituted carrier-free cytokine or phosphate buffered saline (PBS; as vehicle control) under the skin of the abdominal flank of wild-type C57BL/6 mice (three independent, replicate mice per cytokine) in the upper range of previously reported bioactive doses (Methods and Supplementary Table 1). We collected skin-draining lymph nodes (specialized immune organs that integrate signals from surrounding tissues) 4 h after injection, one of the earliest time points at which the majority of the transcriptome responds to immune stimuli^15,16^. We then processed the lymph nodes using an optimized protocol for viable cell recovery, balanced cell-type representation and high-throughput sample multiplexing (Methods). Data quality, including batch-to-batch consistency, was strictly experimentally controlled and computationally verified (Methods). Cells were profiled using a droplet-based system (10x Genomics) to generate high-quality single-cell transcriptomes for 386,703 cells (Fig. 1b and Extended Data Figs. 1 and 2).

After partitioning cells into global clusters, we observed that most cells were segregated by cell-type identity rather than stimulation conditions (Fig. 1b, Extended Data Fig. 1b and Supplementary Table 2). Although cytokine-treated cells did not typically form distinct clusters, they were often separated from PBS controls within each cell-type cluster (Extended Data Fig. 1b,c). After manual inspection to ensure the accuracy of cell-type identification, more than 20 cell types were identified. These corresponded to B cell, CD4^+^ T cell, CD8^+^ T cell, γδ T cell, regulatory T (T_reg_) cell, natural killer (NK) cell, innate lymphoid cell (ILC), plasmacytoid dendritic cell (pDC), conventional dendritic cell type 1 (cDC1), cDC2, migratory DC (MigDC), Langerhans cell^17^, extrathymic Aire-expressing cell (eTAC)^18^, macrophage (*Marco*^+^ or *Lyz1*^+^), monocyte, neutrophil, mast cell, and a small number of less abundant cell types, including basophil, blood endothelial cell (BEC), lymphatic endothelial cell (LEC) and fibroblastic reticular cell (FRC) (Fig. 1b and Extended Data Fig. 1d,e). The frequencies of most cell types remained stable after stimulation, with the notable exception being a significant increase in monocyte fractions in lymph nodes after certain cytokine treatments (Extended Data Fig. 1f,g). To identify cytokine signatures, we computed the significantly differentially expressed genes (DEGs) in response to cytokine treatment in each cell type (Methods and Supplementary Table 3). We identified an average of 51 DEGs (span of 0–1,510) per cytokine–cell type combination, and the majority (72%) of the DEGs responding to cytokines were upregulated rather than downregulated. We verified that the transcriptomic signatures were consistent across replicate animals (Extended Data Fig. 2b) and that every lymph node cell type was able to access the injected cytokines (Extended Data Fig. 2c). We also confirmed robust upregulation of well-established cytokine-responsive genes, such as *Tnfaip3* in response to TNF, *Il4i1* in response to IL-4, and *Isg15* and other interferon-stimulated genes (ISGs) in response to IFNβ (Fig. 1c and Supplementary Table 3).

To chart the immune cell responses to each cytokine, we created a map that quantified the global transcriptomic changes between cytokine-treated and PBS-treated cells for each cell type (Fig. 1d and Methods). The map captured well-known cellular targets of cytokines, such as NK cells responding to IL-2, IL-12, IL-15 and IL-18, and many less characterized responses. Certain cytokines, such as IFNα1, IFNβ, IL-1α, IL-1β, IL-18, IL-36α, IL-15 and TNF, induced strong changes in gene expression in nearly all cell types. Some cytokines preferentially targeted one lineage (for example, IL-21 affected the lymphoid lineage, whereas IL-3 affected the myeloid lineage) or a subset of cell types. The transcriptomic responses of each major immune cell type to each major cytokine constituted an in vivo dictionary of immune responses, which we term the Immune Dictionary.

**Fig. 2:**
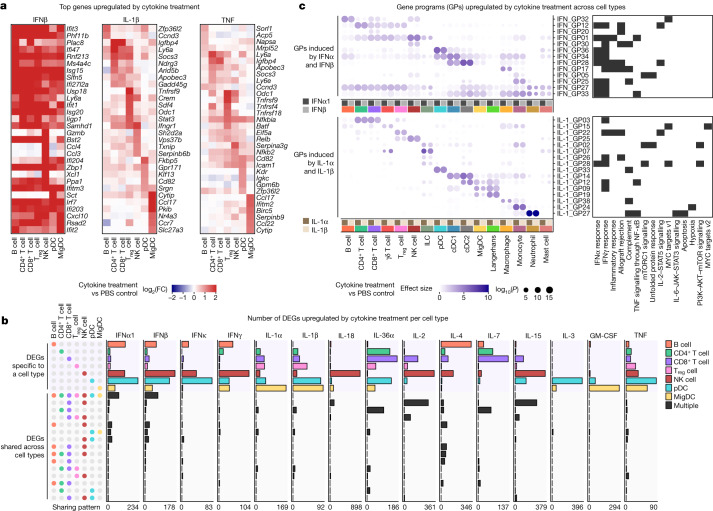
Cytokines induce cell-type-specific transcriptomic responses. **a**, Heatmaps of the top DEGs per cell type in response to IFNβ, IL-1β and TNF relative to PBS controls. Colour gradient represents log_2_(FC) (capped at twofold) in comparison with PBS treatment for the respective cell type. **b**, Number of DEGs (log_2_(FC) > 0.3 and FDR-adjusted *P* < 0.05, two-sided Wilcoxon rank-sum test) following each cytokine treatment, grouped by sharing pattern, either specifically overexpressed by one cell type (top) or shared by two or more cell types (bottom). Cell-type combinations with the most shared genes (>16 DEGs in any given treatment) are shown. A maximum of 100 cells per cytokine treatment for each of the 7 representative cell types were sampled to ensure comparability across cell types for this analysis. The *x* axes span from 0 to the highest DEG counts. **c**, Upregulated GPs following IFNα1 and IFNβ (top) or IL-1α and IL-1β (bottom) treatment with respect to PBS control. GPs that are significantly upregulated between cytokine and PBS treatment (effect size > 1 and FDR-adjusted *P* < 0.01, two-sided Wilcoxon rank-sum test) in any cell type are shown. Significant GPs (FDR < 0.05) for each cell type are represented as circles, with the circle size indicating significance and the colour representing the effect size (capped at 10). Representative enriched biological processes (FDR-adjusted *P* < 0.05; black tiles) for the top-weighted genes in each GP are shown. Source data

## Cell-type-specific cytokine responses

**Extended Data Fig. 3:**
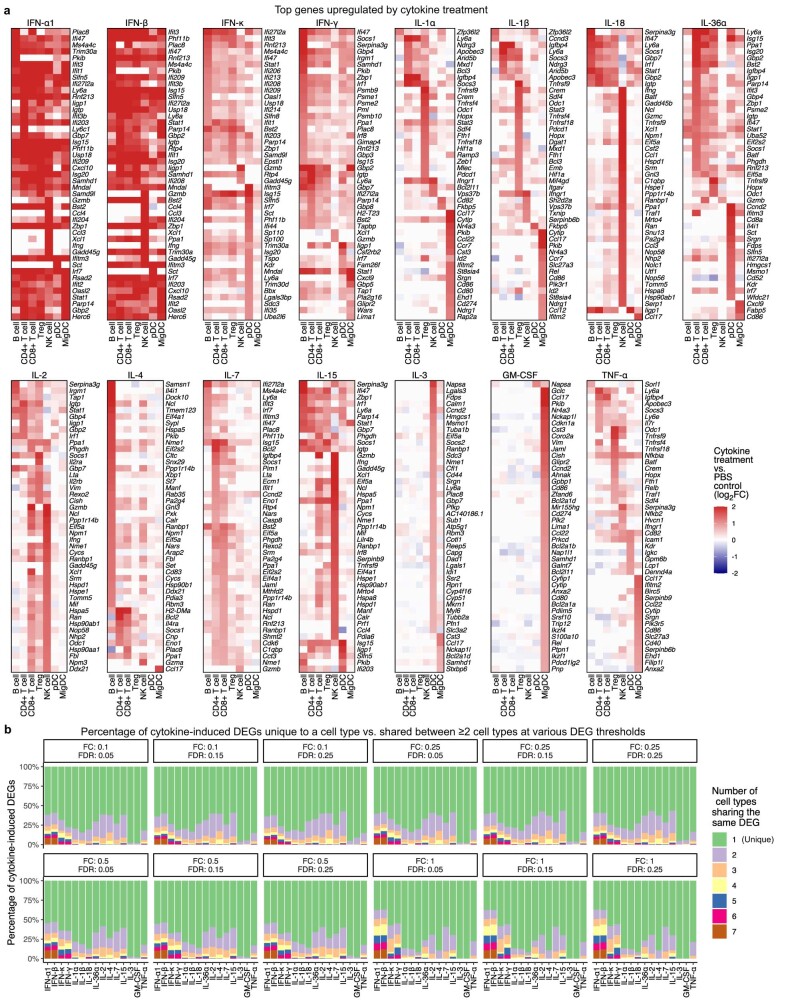
Cell type-specific gene expression changes induced by IFN-α1, IFN-β, IFN-κ, IFN-γ, IL-1α, IL-1β, IL-18, IL-36α, IL-2, IL-4, IL-7, IL-15, IL-3, GM-CSF, and TNF-α. **a**, Gene expression heatmaps illustrating top genes upregulated by cytokine treatment compared to PBS treatment in any of the cell types shown. All genes shown have at least log_2_FC > 0.3 and FDR < 0.05 in one or more cell types. Color gradient indicates average log_2_FC relative to PBS treatment of the same cell type. Genes expressed in fewer than 10% of cells in both cytokine and PBS treatment conditions are denoted as no change. **b**, Percentage of upregulated DEGs exclusive to a single cell type (green) or shared among two or more cell types (other colors) after cytokine treatment at various DEG cutoff thresholds. Each box shows a different log_2_FC and FDR threshold for defining DEGs. This is an extension of the analysis in Fig. 2b, showing consistency of the cell type-specific effects irrespective of DEG cutoffs. Source data

We performed a cytokine-centric analysis of this dictionary to explore how different cell types respond to the same cytokine. Gene expression heatmaps of top upregulated genes in response to IFNβ, IL-1β and TNF revealed that cytokines induced cell-type-specific gene expression changes (Fig. 2a). This observation was consistent across 15 cytokines that induced strong transcriptomic changes in a large number of cell types shown in Fig. 1d, including IL-1α, IL-36α and IL-7 (Extended Data Fig. 3a). We computed the number of upregulated genes that were either specific to a cell type or shared among multiple cell types (Fig. 2b and Supplementary Table 4). Most of the upregulated genes in response to a particular cytokine were specific to one cell type regardless of thresholds for defining DEGs (Extended Data Fig. 3b). Some cytokines induced substantial changes on one or a small number of cell types, such as IL-18 on NK cells, IL-3 on pDCs and GM-CSF on MigDCs. The most shared DEGs were for IFNα1 and IFNβ (across all cell types), IL-4 (across several combinations of cell types), and IL-2, IL-15 and IL-18 (all three of which induced cytotoxic genes in CD8^+^ T cells and NK cells).

To represent complex cytokine responses across cell types in a compact manner, we identified gene programmes (GPs) that consisted of co-expressed genes that became upregulated as a group in response to cytokines (Fig. 2c, Supplementary Fig. 1 and Supplementary Table 5). IFNα1 and IFNβ, as related cytokines, induced highly similar responses to each other, as did IL-1α and IL-1β. IFNα1 and IFNβ, as expected, induced common antiviral GPs across almost all cell types (GP numbers GP27, GP33 and GP34) but also some lineage-specific and cell-type-specific programmes. By contrast, IL-1α and IL-1β, pro-inflammatory cytokines with many known functions in the activation of both innate and adaptive immune cells^19^, induced highly cell-type-specific GPs with a diverse set of enriched biological processes (Fig. 2c). These GPs seemed to enhance the known functions of several of these cell types, including: (1) neutrophils upregulating chemokine and inflammatory genes such as *Cd14* (GP27), consistent with their role as first responders; (2) MigDCs and Langerhans cells upregulating migration programmes including *Ccr7* (GP12); and (3) T_reg_ cells inducing *Hif1a* and *Ctla4* that can mediate immune suppression (GP22). Our results illustrate how the type I interferon response includes a common and autonomous viral defence programme, while IL-1α and IL-1β trigger a coordinated multicellular response composed of highly cell-type-specific functions.

**Extended Data Fig. 4:**
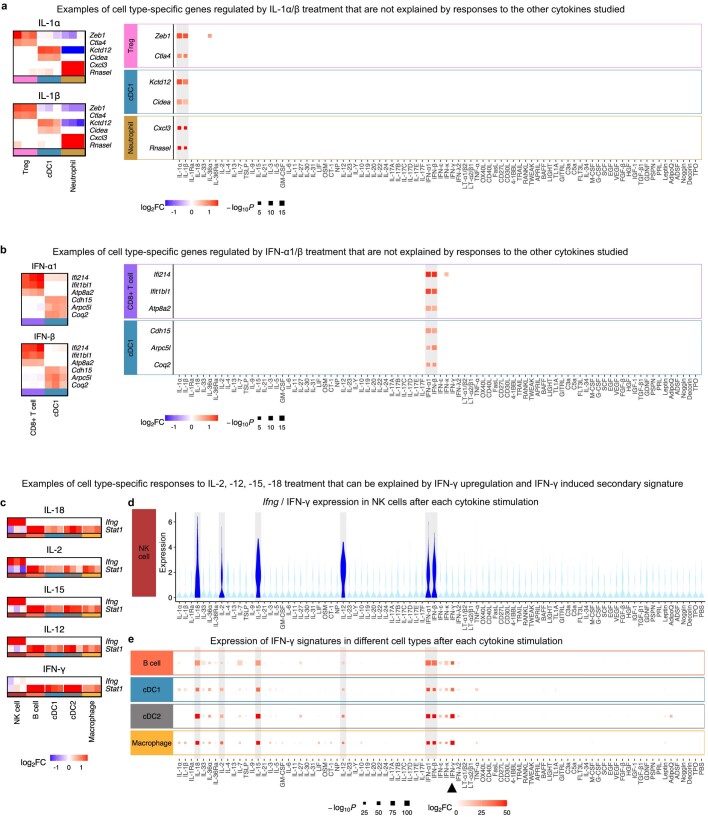
Cell type-specific responses to a cytokine can be exclusive to the cytokine or can be attributed to secondary effects from induced cytokines. **a-b**, Examples of cell type-specific responses to a cytokine that are not observed in the responses to the other cytokines studied. **a**, Examples of cell type-specific gene regulation in response to IL-1α/β and exclusively to IL-1α/β. Left, Heatmaps showing differential expression of IL-1α/β-regulated genes relative to PBS treatment per cell type, highlighting cell type-specific responses to the same cytokines. Three independent mice for each cytokine treatment are shown in adjacent columns. Right, for the IL-1α/β-induced cell type-specific DEGs in the heatmaps, the plots show whether they can be induced by any other of the 86 cytokine stimulations. Color of square, log_2_ fold change in each cytokine treatment relative to PBS; size of square, –log_10_ transformed FDR-adjusted *P*-value obtained from two-sided Wilcoxon rank-sum test. Results with FDR < 0.05 and log_2_FC > 0.5 are shown. IL-1α/β treatments are highlighted in gray. Cell types in columns on the left and rows on the right follow the same color code. Note that both IL-36α and IL-1α/β are proinflammatory cytokines in the IL-1 cytokine family. **b**, Examples of cell type-specific gene regulation in response to IFN-α1/β and exclusively to IFN-α1/β; following the same visualization as in **a**. Note that both IFN-κ and IFN-α1/β are type I interferons and share receptors. **c-e**, Examples of cell type-specific responses to a cytokine that can be attributed to secondary effects from induced cytokines. **c**, Heatmaps showing DEGs in response to IL-2, IL-12, IL−15, IL−18 relative to PBS treatment, highlighting different responses to the same cytokine in NK cells vs. other cell types. Heatmap for IFN-γ treatment is included as a comparison. *Stat1* is known to be regulated by IFN-γ signaling. **d**, Violin plot showing *Ifng* gene expression in NK cells after each of the 86 cytokine treatments or PBS treatment. Samples with a high expression (>0.8 normalized expression units) are colored dark blue. **e**, Induction of IFN-γ signatures across cell types after each cytokine treatment. The IFN-γ signature is obtained for each cell type from the IFN-γ treatment. Expression of the signature is obtained from summing normalized expression units of all genes in the signature for each cell type. Significant (FDR < 0.01) responses are shown as squares, with color gradient representing average log_2_FC in each cytokine treatment relative to PBS (capped at 50), and size representing –log_10_ transformed FDR-adjusted *P*-value obtained from two-sided Wilcoxon rank-sum test. Cytokines inducing a high expression of *Ifng* are highlighted in gray in **d** and **e**. **d** and **e** are vertically aligned to illustrate that the cytokine treatments inducing an upregulation of *Ifng* in NK cells display a strong IFN-γ signature in other cell types. Source data

Many cell-type-specific responses to a single cytokine were not explained by secondary effects to the other cytokines studied (Extended Data Fig. 4a,b). However, some responses could be attributed to secondary effects to induced cytokines, such as the induction of *Ifng* (which encodes IFNγ) in NK cells by IL-2, IL-12, IL-15 and IL-18, which probably in turn stimulate B cells, DCs and macrophages to strongly express IFNγ signatures (Extended Data Fig. 4c–e). As IL-2, IL-12, IL-15 and IL-18 have been applied as therapies with the intention of activating T cells, our results highlight the importance of considering rapidly induced secondary effects on non-intended cell types due to complex in vivo immune responses to a single cytokine. In summary, our systematic analysis of how different cell types respond to each cytokine provides a molecular map for observed pleiotropic effects of cytokines^20^.

## Cytokine-driven cell polarization states

**Fig. 3:**
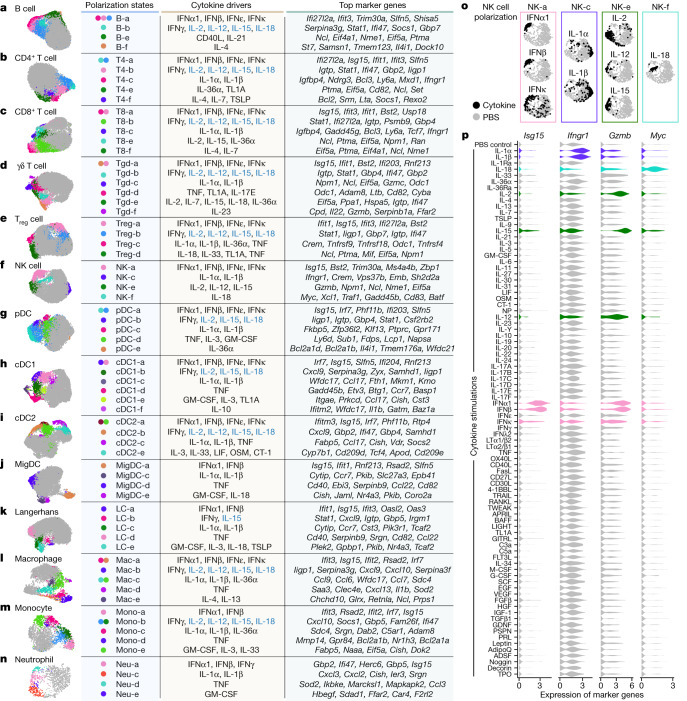
Cytokines drive diverse polarization states in each cell type. **a**–**n**, Uniform manifold approximation and projection (UMAP) plots of cells shown for each cell type. **a**, B cell. **b**, CD4^+^ T cell. **c**, CD8^+^ T cell. **d**, γδ T cell. **e**, T_reg_ cell. **f**, NK cell. **g**, pDC. **h**, cDC1. **i**, cDC2. **j**, MigDC. **k**, Langerhans cell. **l**, *Marco*^+^ macrophage. **m**, Monocyte, **n**, Neutrophil. Coloured circles in the UMAP plots and next to state names correspond to polarization states. Cells coloured grey do not map to polarization states described. Cell polarization state name, single cytokine drivers and top marker genes are shown in the table for each cell type. Cytokine drivers coloured blue are probably indirect inducers. Top marker genes are defined as highly upregulated genes in the polarization state relative to all other cells of the same cell type. Colours in different panels are unrelated. **o**,**p**, Additional views of **f** using NK cells as an example to illustrate polarization-state analyses. **o**, UMAP plots coloured by cytokine or PBS treatment for major cytokine drivers of polarization states shown in **f**. **p**, Violin plots of expression levels of selected marker genes after cytokine or PBS treatment. Colours correspond to the polarization states that the cytokines are most strongly associated. This figure is a summary of the complete landscape for each cell type in Extended Data Figs. 5–8 and Supplementary Figs. 2–11. Source data

**Extended Data Fig. 5:**
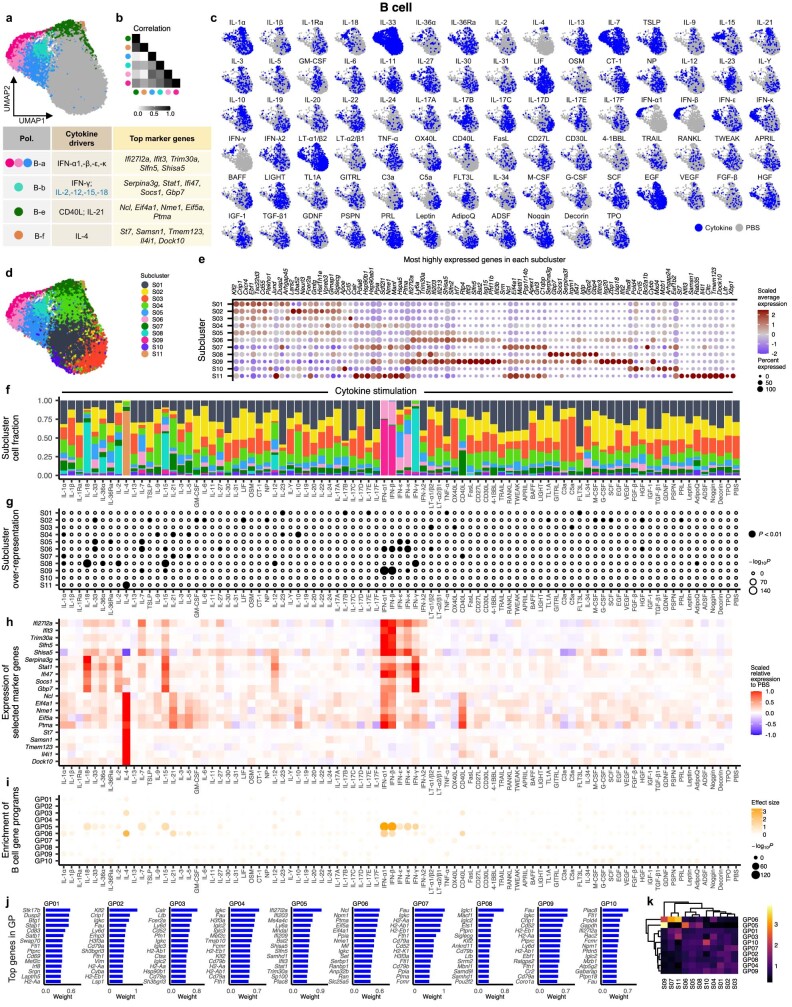
B cell responses to cytokines: polarization states, subclusters, marker gene expression, and gene programs. **a**, Top, UMAP visualization of B cells for all cytokines, colored by polarization states; bottom, table with cell type polarization states (left column), single cytokine drivers (middle column), and top marker genes (right column); reproduced from Fig. 3 for ease of reference. **b**, Pairwise Pearson correlation coefficients between polarization states. **c**, UMAP visualization of B cells shown independently for each cytokine treatment, colored by cytokine treatment (blue) or PBS treatment control (gray). **d**, UMAP visualization of B cells for all cytokine or PBS treatment; cells colored for B cell Louvain subclusters. **e**, Top overexpressed genes in each Louvain subcluster in **d**; color, column-scaled average expression; size of circle, percentage of cells in the subcluster expressing each gene. **f-i**, B cell responses to each cytokine stimulation. **f**, Fraction of cells per subcluster in each cytokine treatment. Colors represent subclusters defined in **d**. **g**, Enrichment of each subcluster in each cytokine treatment; size of circle, Bonferroni-adjusted *P*-value of hypergeometric test relative to PBS; black fills, *P* < 0.01. **h**, Row-normalized relative expression of representative marker genes of each polarization state in cytokine-treated vs. PBS-treated cells. **i**, Enrichment of B cell gene programs obtained from NMF analysis of all B cells in cytokine-treated cells relative to PBS-treated cells; size of circle, FDR-adjusted *P*-value from two-sided Wilcoxon rank-sum test; shade, effect size representing the mean difference in gene program weight. **j**, Top weighted genes in each gene program in **i**. **k**, Average gene program weight in each subcluster. Rows and columns were hierarchically clustered using the complete-linkage method on Euclidean distances. Source data

**Extended Data Fig. 6:**
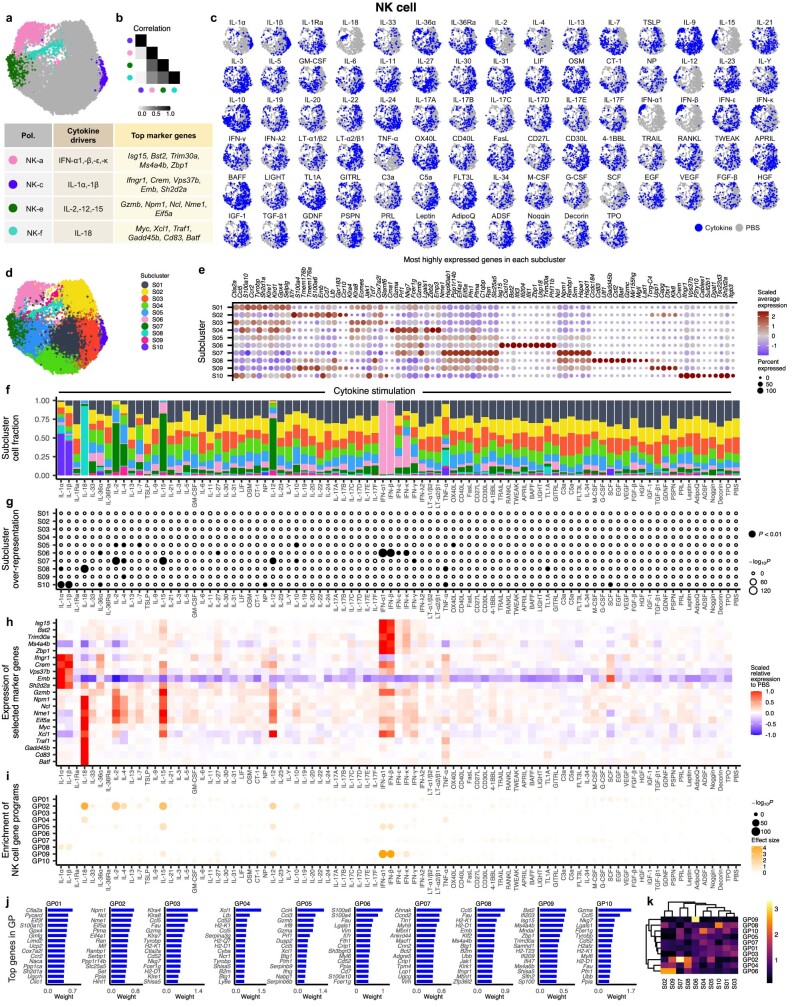
NK cell responses to cytokines: polarization states, subclusters, marker gene expression, and gene programs. **a**, Top, UMAP visualization of NK cells for all cytokines, colored by polarization states; bottom, table with cell type polarization states (left column), single cytokine drivers (middle column), and top marker genes (right column); reproduced from Fig. 3 for ease of reference. **b**, Pairwise Pearson correlation coefficients between polarization states. **c**, UMAP visualization of NK cells shown independently for each cytokine treatment, colored by cytokine treatment (blue) or PBS treatment control (gray). **d**, UMAP visualization of NK cells for all cytokine or PBS treatment; cells colored for NK cell Louvain subclusters. **e**, Top overexpressed genes in each Louvain subcluster in **d**; color, column-scaled average expression; size of circle, percentage of cells in the subcluster expressing each gene. **f-i**, NK cell responses to each cytokine stimulation. **f**, Fraction of cells per subcluster in each cytokine treatment. Colors represent subclusters defined in **d**. **g**, Enrichment of each subcluster in each cytokine treatment; size of circle, Bonferroni-adjusted *P*-value of hypergeometric test relative to PBS; black fills, *P* < 0.01. **h**, Row-normalized relative expression of representative marker genes of each polarization state in cytokine-treated vs. PBS-treated cells. **i**, Enrichment of NK cell gene programs obtained from NMF analysis of all NK cells in cytokine-treated cells relative to PBS-treated cells; size of circle, FDR-adjusted *P*-value from two-sided Wilcoxon rank-sum test; shade, effect size representing the mean difference in gene program weight. **j**, Top weighted genes in each gene program in **i**. **k**, Average gene program weight in each subcluster. Rows and columns were hierarchically clustered using the complete-linkage method on Euclidean distances. Source data

**Extended Data Fig. 7:**
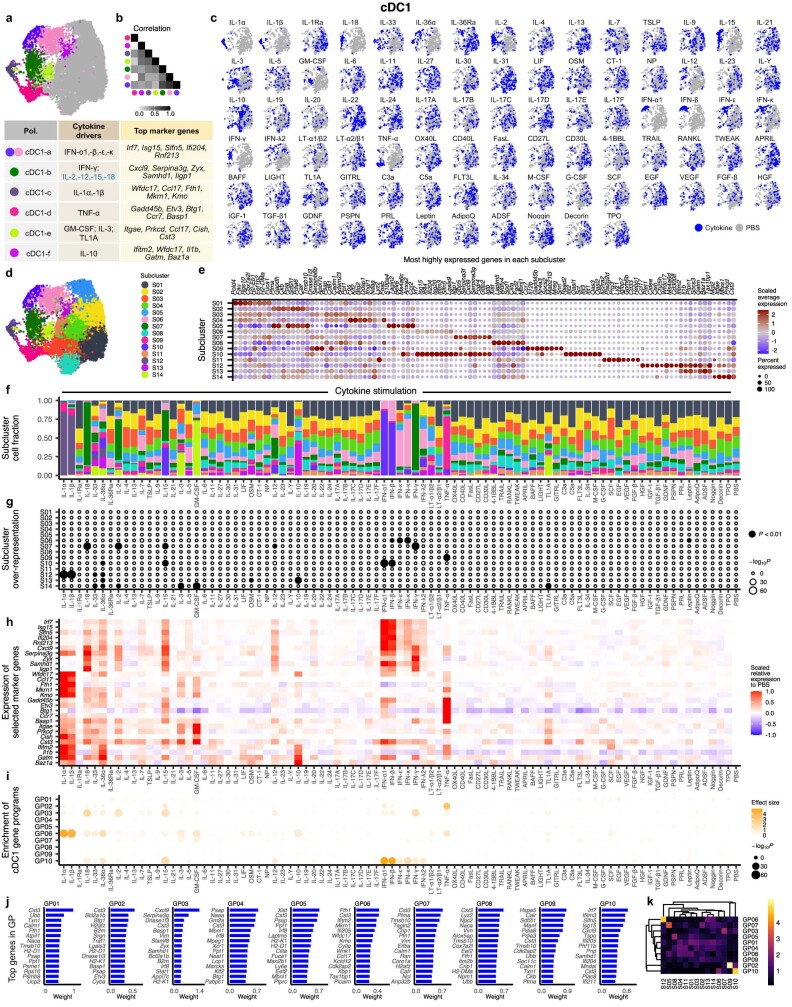
cDC1 responses to cytokines: polarization states, subclusters, marker gene expression, and gene programs. **a**, Top, UMAP visualization of cDC1 cells for all cytokines, colored by polarization states; bottom, table with cell type polarization states (left column), single cytokine drivers (middle column), and top marker genes (right column); reproduced from Fig. 3 for ease of reference. **b**, Pairwise Pearson correlation coefficients between polarization states. **c**, UMAP visualization of cDC1 cells shown independently for each cytokine treatment, colored by cytokine treatment (blue) or PBS treatment control (gray). **d**, UMAP visualization of cDC1 cells for all cytokine or PBS treatment; cells colored for cDC1 Louvain subclusters. **e**, Top overexpressed genes in each Louvain subcluster in **d**; color, column-scaled average expression; size of circle, percentage of cells in the subcluster expressing each gene. **f-i**, cDC1 responses to each cytokine stimulation. **f**, Fraction of cells per subcluster in each cytokine treatment. Colors represent subclusters defined in **d**. **g**, Enrichment of each subcluster in each cytokine treatment; size of circle, Bonferroni-adjusted *P*-value of hypergeometric test relative to PBS; black fills, *P* < 0.01. **h**, Row-normalized relative expression of representative marker genes of each polarization state in cytokine-treated vs. PBS-treated cells. **i**, Enrichment of cDC1 gene programs obtained from NMF analysis of all cDC1 cells in cytokine-treated cells relative to PBS-treated cells; size of circle, FDR-adjusted *P*-value from two-sided Wilcoxon rank-sum test; shade, effect size representing the mean difference in gene program weight. **j**, Top weighted genes in each gene program in **i**. **k**, Average gene program weight in each subcluster. Rows and columns were hierarchically clustered using the complete-linkage method on Euclidean distances. Source data

**Extended Data Fig. 8:**
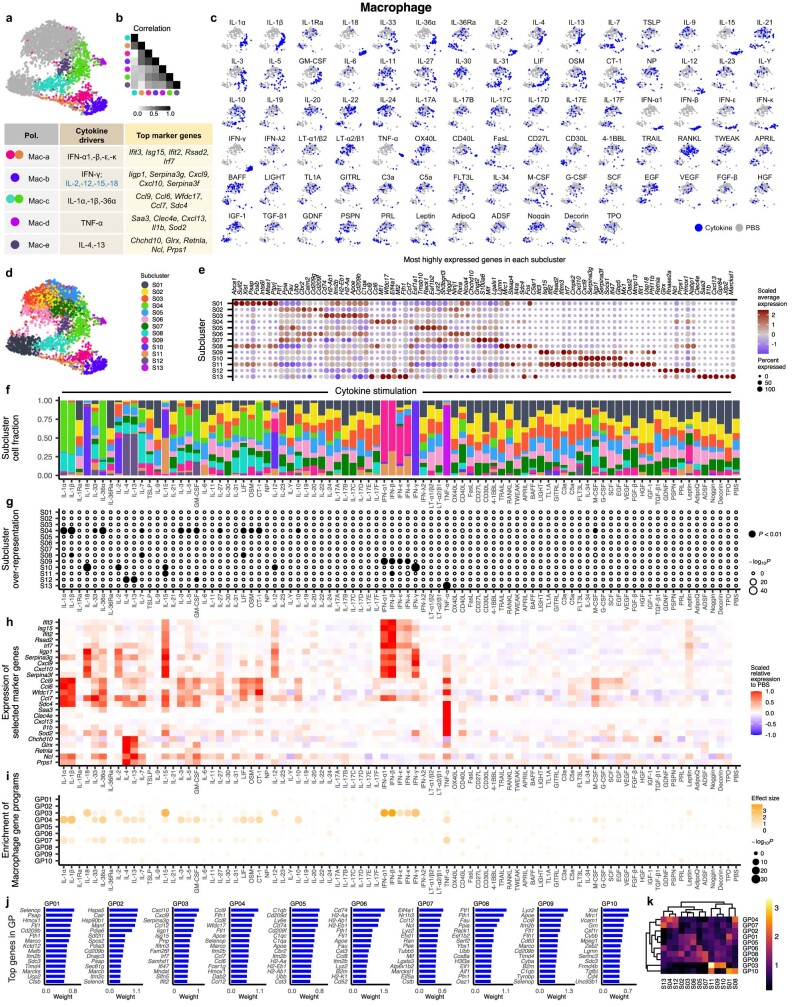
Macrophage (*Marco*+) responses to cytokines: polarization states, subclusters, marker gene expression, and gene programs. **a**, Top, UMAP visualization of *Macro*+ macrophages for all cytokines, colored by polarization states; bottom, table with cell type polarization states (left column), single cytokine drivers (middle column), and top marker genes (right column); reproduced from Fig. 3 for ease of reference. **b**, Pairwise Pearson correlation coefficients between polarization states. **c**, UMAP visualization of macrophages shown independently for each cytokine treatment, colored by cytokine treatment (blue) or PBS treatment control (gray). **d**, UMAP visualization of macrophages for all cytokine or PBS treatment; cells colored for macrophage Louvain subclusters. **e**, Top overexpressed genes in each Louvain subcluster in **d**; color, column-scaled average expression; size of circle, percentage of cells in the subcluster expressing each gene. **f-i**, Macrophage responses to each cytokine stimulation. **f**, Fraction of cells per subcluster in each cytokine treatment. Colors represent subclusters defined in **d**. **g**, Enrichment of each subcluster in each cytokine treatment; size of circle, Bonferroni-adjusted *P*-value of hypergeometric test relative to PBS; black fills, *P* < 0.01. **h**, Row-normalized relative expression of representative marker genes of each polarization state in cytokine-treated vs. PBS-treated cells. **i**, Enrichment of macrophage gene programs obtained from NMF analysis of all macrophages in cytokine-treated cells relative to PBS-treated cells; size of circle, FDR-adjusted *P*-value from two-sided Wilcoxon rank-sum test; shade, effect size representing the mean difference in gene program weight. **j**, Top weighted genes in each gene program in **i**. **k**, Average gene program weight in each subcluster. Rows and columns were hierarchically clustered using the complete-linkage method on Euclidean distances. Source data

We next performed a cell-type-centric analysis of the dictionary to identify cell states induced by cytokines. Cytokines are major drivers of immune cell polarization, with a classic example being distinct cytokines driving macrophages into pro-inflammatory M1-like or reparative M2-like states^21^. However, the polarization states of many immune cell types have not been comprehensively characterized, and even macrophage polarization is more complex than the M1/M2 dichotomy^22^. Gene expression profiling can simultaneously measure the entire transcriptome and has been particularly useful for defining cell states driven by environmental cues^13,23,24^. Here we leveraged the dictionary to systematically identify single cytokine-induced cell polarization states. We subclustered each immune cell type and defined 66 major polarization states as subclusters significantly enriched for cytokine-treated relative to PBS-treated cells and expressing meaningful biological programmes (Methods and Fig. 3, with the complete landscape in Extended Data Figs. 5–8, Supplementary Figs. 2–11 and Supplementary Tables 6–8). Each polarization state was induced by one or a handful of dominant cytokine drivers (Fig. 3a–n, Extended Data Figs. 5f,g, 6f,g, 7,f,g and 8f,g and Supplementary Figs. 2f,g, 3f,g, 4f,g, 5f,g, 6f,g, 7f,g, 8f,g, 9f,g, 10f,g and 11f,g).

We examined macrophages and monocytes to see whether this approach uncovers previously established polarization states (Fig. 3l,m, Extended Data Fig. 8 and Supplementary Fig. 10). As expected from previous studies^13^, IFNγ induced a ‘Mac-b’ state that overexpresses M1-associated pro-inflammatory genes (for example, *Cxcl9* and *Cxcl10*). IL-4 and IL-13 induced a distinct ‘Mac-e’ state that was not marked by these pro-inflammatory genes but by *Chchd10*, *Glrx* and *Retnla*. We also confirmed previous findings that monocytes had higher *Il1b* expression when treated with IL-1α, IL-1β or IL-36α (Supplementary Fig. 10h), thereby potentially creating an inflammatory feed-forward loop^19^. In addition, IFNα1, IFNβ and TNF triggered other polarization states.

NK cells are known for their cytotoxic functions, but other functions are still being discovered^25^. As expected from previous knowledge, IL-2, IL-12 and IL-15 induced a state with increased expression of cytotoxic genes, and type I interferons induced the expression of both cytotoxic genes and ISGs (Fig. 3f,o,p and Extended Data Fig. 6). IL-1α and IL-1β induced a distinct ‘NK-c’ state with low correlation with other NK cell states and lacking overexpression of cytotoxic molecules (Extended Data Fig. 6b). However, in this state, *Ifngr1* was upregulated, which potentially enhances NK cell activation by IFNγ (Fig. 3p, Extended Data Fig. 6 and Supplementary Fig. 12). Although IL-18 is known to activate NK cells, we found that IL-18-activated NK cells, predominantly in the ‘NK-f’ state, displayed markedly different properties than NK cells activated by IL-2, IL-12 or IL-15. IL-18 triggered the upregulation of more than 1,000 genes (Fig. 2b and Supplementary Table 3), an order of magnitude more than cells stimulated with other cytokines. This had a partial overlap with the IL-2, IL-12, IL-15 and interferon states, including *Gzmb* and *Xcl1*. Compared with PBS treatment, the IL-18-induced state was strongly enriched in biosynthetic processes, including the induction of *Myc* (which controls growth and proliferation), immune processes such as maturation of myeloid cells (*Csf2* (which encodes GM-CSF)), recruitment of DCs (*Xcl1*), cytotoxicity (*Gzmb*) and regulators of differentiation (*Kit* and *Batf*) (Fig. 3p, Extended Data Fig. 6 and Supplementary Fig. 12). IL-18 has shown promise in preclinical studies of cancer immunotherapy^26^ and can activate T cells, NK cells and other cell types^27^. This unique and strong NK cell response to IL-18 suggests a polyfunctional role for the IL-18–NK cell axis in the immune system.

B cells were polarized by IL-4 to a distinct *Il4i1*^+^ state and by CD40L or IL-21 to a proliferating phenotype **(**Fig. 3a and Extended Data Fig. 5). Similar to their effect on NK cells, IL-1α and IL-1β induced *Ifngr1* upregulation in T cell subsets (Supplementary Figs. 2h and 3h). γδ T cells exhibited diverse polarization states, including a distinct ‘Tgd-f’ state induced by IL-23 that overexpresses *Il22*, and a ‘Tgd-d’ state induced by TNF, TL1A or IL-17E that overexpresses *Odc1* (Fig. 3d and Supplementary Fig. 4). While prior studies of lymphocyte differentiation states have been in the context of TCR or BCR stimulation, our findings demonstrate that resting lymph node B cells and T cells can also be polarized by cytokines to express diverse GPs.

We observed shared polarization states and cytokine drivers in MigDCs and Langerhans cells (Fig. 3j,k and Supplementary Figs. 8 and 9). TNF uniquely increased the proportion of cells in a state marked by *Cd40* and *Ccl22* expression, thereby potentially enhancing antigen presentation and interaction with T cells through the CCR4–CCL22 axis. IL-1α and IL-1β increased the expression of *Ccr7*, and GM-CSF and IL-1 family cytokines induced the upregulation of *Nr4a3*, which has a key role in DC migration^28^. These results suggest that TNF can trigger local inflammation and DC–T cell interactions, and that the IL-1 family cytokines can boost DC migration to prime T cell responses in lymph nodes.

**Extended Data Fig. 9:**
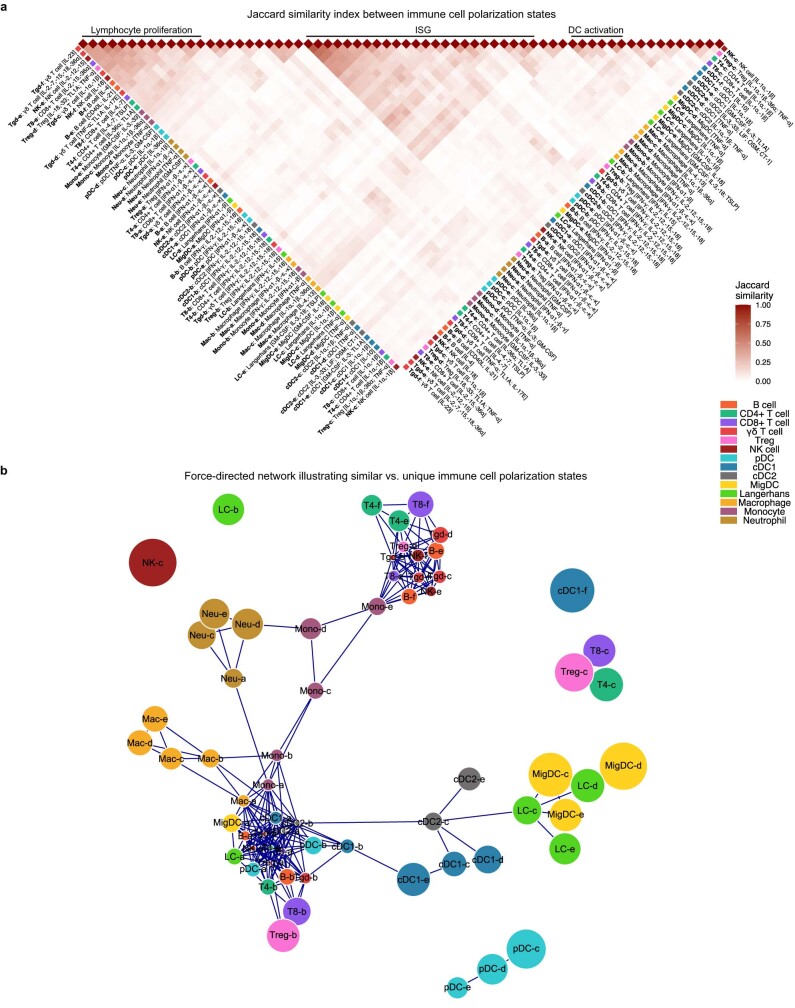
A comparative global view of the 66 major polarization states across immune cell types. **a**, Heatmap showing pairwise Jaccard similarity index between immune cell polarization states defined in Fig. 3, Extended Data Figs. 5–8, and Supplementary Figs. 2–11. Jaccard similarity index is defined based on genes with >0.5 log_2_ fold difference in each polarization state compared to PBS-treatment of the same cell type. Cell types are marked by colors on the edges of the heatmap. Cytokine drivers are indicated in square brackets. Groups of similar polarization states are annotated above the heatmap. **b**, Force-directed graph visualization of Jaccard similarity index between immune cell polarization states to highlight unique polarization states. Vertices represent polarization states. A line connects a pair of vertices that have Jaccard similarity index > 0.15. Unconnected states are randomly positioned in the force-directed graph. A larger circle represents a more unique polarization state, based on a lower median Jaccard similarity index with other polarization states. Cell types are marked by colors indicated in **a**. Source data

Overall, our reference map of cytokine-driven cellular polarization states reveals the plasticity of all immune cell types. These polarization states may be shared across cell types, such as type I interferons inducing ISG I states and type II interferons inducing ISG II states in each cell type (Extended Data Fig. 9a), or specific for one cell type, such as ‘cDC1-f’ induced by IL-10 to cDC1 (Extended Data Fig. 7), ‘pDC-e’ induced by IL-36α to pDCs (Supplementary Fig. 6) and ‘NK-c’ induced by IL-1α or IL-1β to NK cells (Extended Data Fig. 9b).

## Cytokine production–response map

**Fig. 4:**
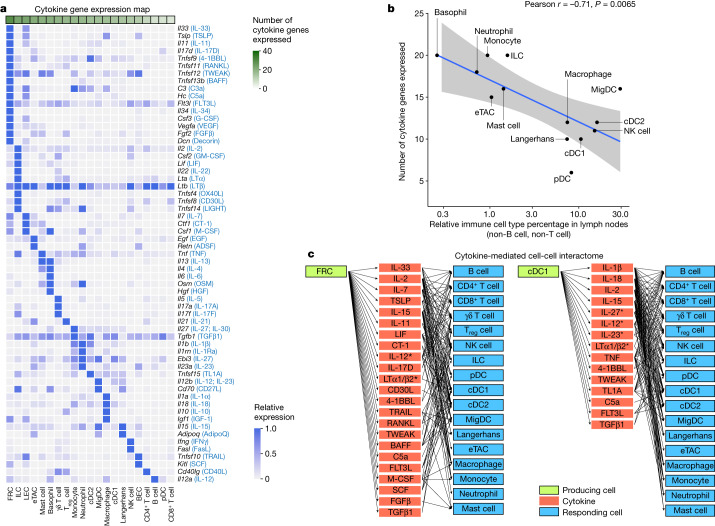
Cytokine production map by cell type. **a**, Heatmap of row-normalized gene expression of the 86 cytokines studied. Protein names encoded by the genes are in parentheses. **b**, Scatter plot showing the abundance of each cell type (log_10_ scaled) in lymph nodes in PBS-treated controls compared with the number of cytokine genes expressed (calculated from an equal number of cells per cell type; threshold of detection = 0.1). Smoothed conditional means and 95% confidence intervals from a fitted linear model are shown. Pearson correlation coefficient and its associated *P* value obtained from two-sided *t*-test are shown. **a** included all cells in the study, whereas **b** sampled an equal number of cells per cell type to ensure comparability across cell types. **c**, Cytokine-mediated cell–cell interactome shown for FRC and cDC1 cells (complete interactome presented in Extended Data Fig. 11). Asterisks indicate multimeric cytokines. Source data

**Extended Data Fig. 10:**
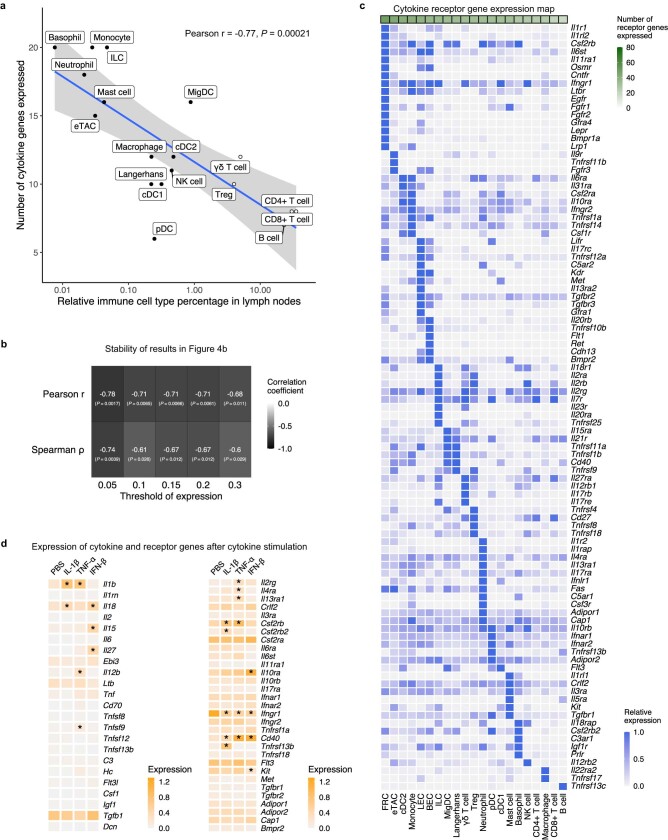
A map of cytokine receptor expression by cell type and additional information on the cytokine production map. **a**, Correlation between cell type abundance and number of distinct cytokine genes expressed; added B cells and T cells (hollow circles) to Fig. 4b. B cell and T cell abundances are estimates integrated from literature, all other cell types are obtained from PBS-treated conditions in lymph nodes from our dictionary. Smoothed conditional means and 95% confidence intervals from a fitted linear model are shown. **b**, Robustness analysis for Fig. 4b; showing correlations between the abundance of each cell type and the numbers of distinct cytokine genes expressed in the cell type under various cutoff thresholds for a cytokine gene to be considered expressed. Both Pearson and Spearman correlation coefficients are shown. **c**, A map of cytokine receptor expression by cell type. The map includes signaling receptors, decoy receptors, as well as receptors that form complexes with cytokines. The values are normalized to the maximum expression in each row. **d**, Expression of cytokine (left) or receptor (right) genes in cDC1s following PBS or cytokine treatment. * represents FDR-adjusted *P* < 0.05 for significant change in expression relative to PBS control. Source data

To better understand the cell–cell communication carried out by each cell type, we identified the production sources of each cytokine by quantifying the corresponding transcript levels averaged across all baseline and cytokine-stimulated conditions to account for stimulation-induced expression (Fig. 4a and Supplementary Table 9). FRCs expressed the highest number of distinct cytokines, consistent with previous findings regarding their heterogeneity and maintenance functions in various immune and non-immune compartments^29^. Other rare cell types in lymph nodes, such as basophils and ILCs, also expressed a large number of cytokines. There was an inverse correlation between the abundance of an immune cell type and the number of cytokines that the cell type produced (Fig. 4b and Extended Data Fig. 10a), with cytokine production calculated using the same number of cells per cell type to ensure comparability. These findings, which were robust against different values of analysis parameters (Extended Data Fig. 10b), suggest that rarer cell types are crucial players in immune cell–cell communication networks despite their low numbers.

**Extended Data Fig. 11:**
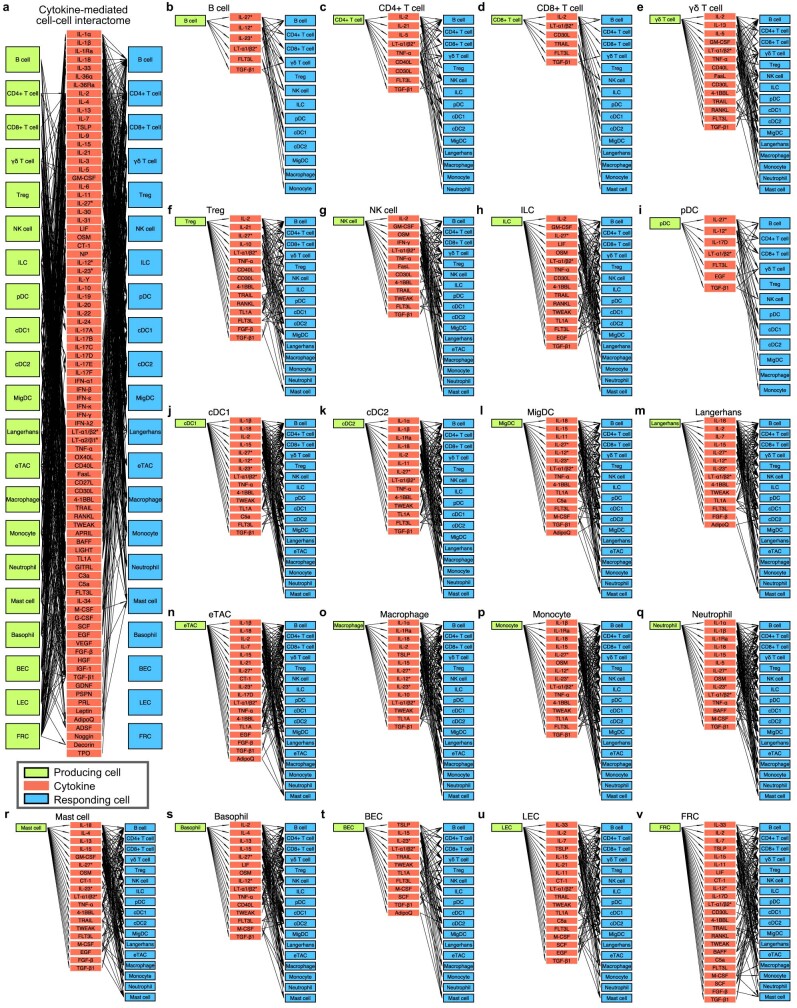
A draft network of cytokine-mediated cell-cell interactome. **a**, An interactome network showing cell-cell communication potential based on cytokine expression and the impact of cytokine on each cell type. Lime box, source nodes or cell types secreting cytokines; red box, cytokines mediating the communication; blue box, sink nodes or cell types responding to cytokines. A path is established between source and sink cell types through a cytokine if the source cell type produces the cytokine (normalized expression > 0.1) and the sink cell type shows a significant response to the cytokine (>10 DEGs in the cytokine signatures). Asterisks indicate heteromeric cytokines or cytokine complexes. Rare cell types, including basophils, BECs, LECs, and FRCs, were not analyzed for the response, but were aggregated across treatment conditions to generate the production map. **b-v**, The interactome using same conventions as in **a** plotted separately by source node for ease of visualization, shown for **b**, B cell; **c**, CD4+ T cell; **d**, CD8+ T cell; **e**, γδ T cell; **f**, Treg; **g**, NK cell; **h**, ILC; **i**, pDC; **j**, cDC1; **k**, cDC2; **l**, MigDC; **m**, Langerhans cell; **n**, eTAC; **o**, macrophage; **p**, monocyte; **q**, neutrophil; **r**, mast cell; **s**, basophil; **t**, BEC; **u**, LEC; **v**, FRC. **j** and **v** are reproduced from Fig. 4c. Source data

On the basis of cytokine production levels inferred from the abundance of transcripts encoding each cytokine and of cytokine responses obtained from the global analysis (Fig. 1d), we built a cell–cell interactome charting available cell–cell communication channels in the immune system (Fig. 4c, Extended Data Fig. 11 and Supplementary Table 10). Our data indicated that FRCs can influence nearly every cell type through a multitude of produced cytokines (Fig. 4c, left). cDC1 cells can also affect almost all cell types, but through a smaller number of cytokines, most prominently through IL-1β, which affects many cells (Fig. 4c, right). A global view of the network showed that most cell types can affect almost every other cell type through at least one cytokine (with the exception of B cells and T cells, which are not stimulated with antigens used in our study), demonstrating a high level of interconnectivity in the immune system (Supplementary Fig. 13).

In addition, we created a cytokine–receptor expression map per cell type (Extended Data Fig. 10c and Supplementary Table 9). Cytokine treatment induced changes in cytokine or receptor expression, such as the upregulation of *Cd40* in cDC1 cells in response to TNF and IFNβ (Extended Data Fig. 10d), which potentially sensitizes cells to subsequent response to other cytokines. Some cytokines induced responses even in the absence of highly expressed receptors, such as IL-1α and IL-1β affecting T cells, NK cells and DCs (Supplementary Fig. 12j and Supplementary Table 10). This effect could be due to insensitive detection of receptor transcripts, rapid secondary effects to molecules induced by the cytokine or unknown receptors. Thus, our cell–cell interactome reveals diverse ways by which cells can interact with one another in vivo through the cytokine network.

## **Immune Response Enrichment Analysis**

The use of transcriptomics to study immune processes and diseases has become standard and has led to the generation of large public datasets^30,31^. However, transcriptomic data do not reveal the factors that trigger the observed cell states and their functions, calling for an approach for inferring cytokine responses and revealing cell–cell communication networks based on cytokine-induced gene expression programmes^32,33^. Most methods to infer cell–cell interactions from transcriptomic data use ligand and receptor expression associations^34,35^. However, receptor expression alone is not an accurate predictor of cytokine responses, as the ligands may not reach the cell or downstream pathways may not be functional. A more precise approach should also consider whether cells express the response signature of a cytokine as defined in our dictionary. Furthermore, there is a need for a computational approach to automatically assess immune cell polarization from transcriptomic data. 

**Fig. 5:**
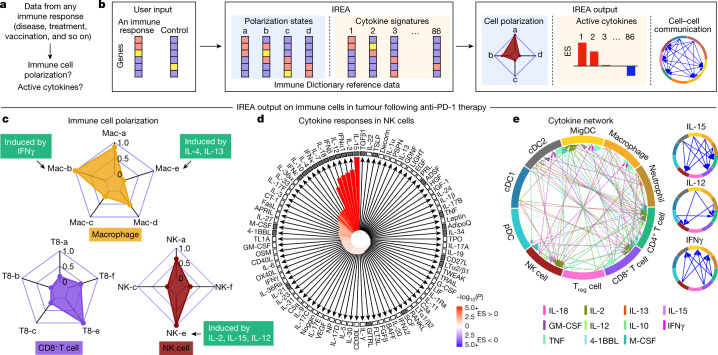
IREA enables the inference of cytokine activities, immune cell polarization and cell–cell communications based on transcriptomic data. **a**, Problem statement for cytokine response inference, **b**, Illustration of the IREA software input and output. Colours in the heatmaps represent different expression values. User input can be gene sets or transcriptome matrices. Blue backgrounds represent cell polarization analysis; orange backgrounds represent cytokine response analysis. **c**–**e**, Examples of IREA analysis output on scRNA-seq data collected from cells in the tumour microenvironment following anti-PD-1 treatment relative to control antibody treatment. **c**, IREA radar plots showing enrichment of macrophage, NK cell and CD8^+^ T cell polarization states described in Fig. 3. Cytokine drivers of selected polarization states are indicated by arrows. **d**, IREA cytokine enrichment plot showing the enrichment score (ES) for each of the 86 cytokine responses in NK cells following anti-PD-1 treatment. Bar length represents the ES, shading represents the FDR-adjusted *P* value (two-sided Wilcoxon rank-sum test), with darker colours representing more significant enrichment (red, enriched in anti-PD-1 treatment, blue, enriched in untreated control). Cytokines with receptors expressed are indicated by black filled boxes. **e**, Inferred cell–cell communication network mediated by cytokines. For ease of identification, cytokines are plotted from left to right in each segment in the same order as the legend. For clarity, individual cytokine plots following the same visualization scheme are shown on the right and in Extended Data Fig. 12c. Source data

To infer immune cell polarization and cytokine responses from any transcriptomic data, we created Immune Response Enrichment Analysis (IREA), which is the companion software for the Immune Dictionary. IREA implements statistical tests to assess the enrichment of either cell polarization or cytokine signatures in transcriptomes, which can then be used to derive cell–cell communication networks that explain observed immune responses (Methods and Fig. 5a,b).

**Extended Data Fig. 12:**
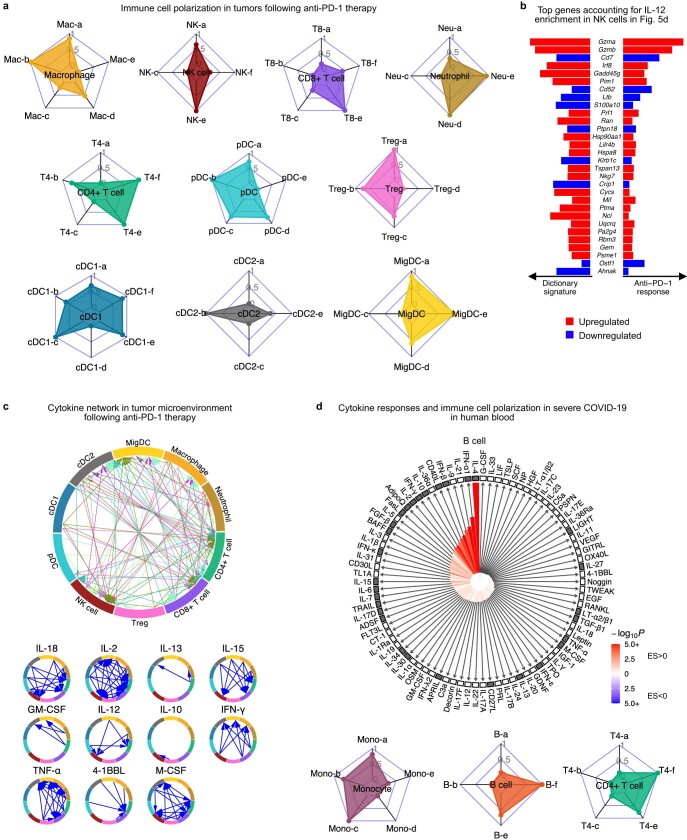
IREA software output on mouse tumor samples following anti-PD-1 treatment and on human blood samples in severe COVID-19. **a-c**, Additional IREA analyses on mouse tumor samples following anti-PD-1 treatment relative to the control. **a**, IREA radar plots showing enrichments of immune cell polarization states described in Fig. 3. **b**, To improve the interpretability of the enrichment scores in Fig. 5d, top genes contributing to the IL-12 enrichment in NK cells are shown. Bar length represents gene expression fold change; left: changes in gene expression in NK cells in response to IL-12 in the Immune Dictionary; right: changes in gene expression in NK cells after anti-PD-1 treatment in the tumor dataset (red: upregulated relative to control; blue: downregulated relative to control). **c**, Inferred cell-cell communication network mediated by cytokines. The plot on the top is reproduced from Fig. 5e for ease of reference. Individual cytokine plots following the same visualization scheme are shown below. **d**, IREA analysis on peripheral blood cells collected from severe COVID-19 patients relative to healthy volunteers. Top, IREA compass plot showing enrichment scores for each of the 86 cytokines in B cells. Bar length represents enrichment score, shade represents FDR adjusted *P*-value (two-sided Wilcoxon rank-sum test), with darker colors representing more significant enrichment (red: enriched in ventilated COVID-19 patients, blue: enriched in healthy control). Cytokines with receptors expressed are indicated by black filled boxes. Bottom, IREA radar plots showing enrichments of immune cell polarization states in monocytes, B cells, and CD4+ T cells. The reference polarization states are described in Fig. 3. Source data

We applied IREA to a published single-cell transcriptomic dataset from immune cells in tumours of mice treated with an anti-PD-1 checkpoint blockade therapy^36^ (Fig. 5c–e and Extended Data Fig. 12a–c). IREA automatically inferred that monocytes and macrophages after treatment polarized into the IFNγ-induced ‘Mac-b’ (M1-like) state and away from the IL-4-induced ‘Mac-e’ state, which is in accordance with the known antitumour properties of IFNγ-polarized M1-like macrophages (Fig. 5c). This method enabled the characterization of immune cells across multiple polarization states on a continuous scale, consistent with recent views that immune cells are not dichotomously polarized^22^. Polarization was also identified in other cell types, including the polarization of NK cells into a cytotoxic ‘NK-e’ state, which can be induced by IL-2, IL-12 and IL-15 (Fig. 5c and Extended Data Fig. 12a). NK cells were enriched for signatures of these cytokines, and IL-12 subunit genes were expressed in DCs and other myeloid cells (Fig. 5d, red bars, Fig. 5e), in agreement with the known role of IL-12 in anti-PD-1 therapy^37^. Among the 86 cytokines, IREA found that the immunosuppressive cytokine TGFβ1 showed the most negative response in anti-PD-1-treated cells compared with untreated cells, consistent with its known role in attenuating the immune enhancement from PD-1 and PD-L1 blockade^38^. These and various other cytokines were produced by and acted on specific cell types in the tumour, which created a cytokine network in the effective response to immunotherapy (Fig. 5d,e). Receptors were expressed for cytokines with inferred responses, but also for some cytokines without responses, highlighting that the presence of receptors is not sufficient for response (Fig. 5d). Applying IREA to severe COVID-19 infection^39^ revealed responses to cytokines in B cells and T cells in severe disease, reflecting known increases in plasma cytokines that regulate lymphocytes (Extended Data Fig. 12d and Supplementary Fig. 14). Our framework therefore enabled us to infer the key secreted factors that trigger observed cellular responses (for example, Fig. 5c,d) and to generate a molecular model of cell–cell interactions (for example, Fig. 5e) that underlie a complex immune response.

## Discussion

Our dictionary of in vivo immune responses to cytokines enabled a high-resolution view of the cytokine network, which showed that the complexity of cytokine responses and plasticity of immune cells are much greater than previously appreciated. Even a single cytokine, such as IL-1β, can trigger distinct responses in each cell type to create a coordinated multicellular immune response. Extending early discoveries of macrophage polarization, we systematically identified cytokine-induced polarization states in each immune cell type, thereby highlighting the general property of immune cells in their plastic responses to environmental cues. We created cytokine response and cytokine–receptor expression maps and used them to derive a cell–cell interactome that illustrated diverse ways that immune cells can interact with one another and the role of rare cell types in immune cell–cell communication. Finally, we introduced IREA, a method for inferring cytokine activities, immune cell polarization and cell–cell communication networks in any immune process for which gene expression data have been collected. Note that because the dictionary was collected at single-cell resolution, one can easily re-analyse the responses in any cell subpopulation of interest. Future directions include studying different cytokine doses, times, biological contexts and combinations of stimuli. In summary, our study created a systematic cell-type-specific dictionary of cytokine responses, providing new insights into cytokine functions and a basis for inferring cell–cell communication networks in any immune response.

## Methods

### Cytokine injection

A list of the cytokines studied and their alternative names are shown in Fig. 1d and Supplementary Table 1. We selected 86 cytokines representing most members of major cytokine families, including the following families: IL-1 (IL-1α, IL-1β, IL-1Ra, IL-18, IL-33, IL-36α and IL-36Ra); common γ-chain/IL-13/TSLP (IL-2, IL-4, IL-13, IL-7, TSLP, IL-9, IL-15 and IL-21); common β-chain (GM-CSF, IL-3 and IL-5); IL-6/IL-12 (IL-6, IL-11, IL-27, IL-30, IL-31, LIF, OSM, CT-1, NP, IL-12, IL-23 and IL-Y); IL-10 (IL-10, IL-19, IL-20, IL-22 and IL-24); IL-17 (IL-17A, IL-17B, IL-17C, IL-17D, IL-17E, IL-17F); interferon (type I: IFNα1, IFNβ, IFNε and IFNκ; type II: IFNγ; and type III: IFNλ2); TNF (LTα1/β2, LTα2/β1, TNF, OX40L, CD40L, FasL, CD27L, CD30L, 4-1BBL, TRAIL, RANKL, TWEAK, APRIL, BAFF, LIGHT, TL1A and GITRL); complement (C3a and C5a); growth factor (FLT3L, IL-34, M-CSF, G-CSF, SCF, EGF, VEGF, FGFβ, HGF and IGF-1). Representative cytokines (TGFβ1, GDNF, persephin (PSPN), prolactin (PRL), leptin, adiponectin (AdipoQ), resistin (ADSF), noggin, decorin and thrombopoietin (TPO)) from other protein families were also included.

Every recombinant mouse cytokine was obtained from at least two separate orders. The endotoxin level was <0.1 ng µg^–1^ of protein for every cytokine per the information from the vendors (Peprotech and R&D). To preserve cytokine activities, carrier-free cytokines were freshly reconstituted according to the manufacturer’s instructions, stored at 4 °C in sterile conditions and used within 28 h after reconstitution. For each cytokine, 5 μg in 100 μl sterile PBS was injected into each animal. Wild-type female C57BL/6 mice were purchased from the Jackson Laboratory and used in studies as 11–15-week-old young adults after resting for at least 1 week in the facility. Mice were maintained on a 12-h light–dark cycle at room temperature (21 ± 2 °C) and 40 ± 10% humidity. Cytokines were injected under the skin (50% subcutaneous, 50% intradermal) bilaterally in the abdominal flank of each mouse. Bilateral skin-draining inguinal lymph nodes were collected 4 h after injection at 6:00–8:00 and pooled for downstream processing. For each of the 86 cytokines, replicate experiments were performed in three independent C57BL/6 mice to ensure reproducibility. As a control, PBS alone was injected into mice for each experimental batch, totalling 14 PBS-injected mice. All experiments were reviewed and approved by the Broad Institute’s Institutional Animal Care and Use Committee.

### Data generation quality assurance

All samples were processed using an optimized experimental pipeline to ensure quality. In particular, batch effects that arise from experiments performed on different days are known to be a major source of artefact in transcriptomic studies. Therefore, batch-to-batch consistency was strictly experimentally ensured and then computationally verified. Specifically, the mice were ordered from the same batch and housed in the same environment. Animals were randomly allocated to the experimental groups. Lymph nodes were collected at 6:00–8:00 in all experiments to exclude the impact of circadian clocks on transcriptomic profiles. Samples were processed fresh in every experiment and were kept on ice during processing whenever possible. The same researchers performed the same steps of the sample processing and sequencing pipeline following the same, highly optimized procedures. The investigators performing animal experiments and RNA sequencing were blinded from each other during data collection. The number of batches was minimized whenever possible. The three replicated mice for each cytokine were processed in different batches to ensure that batch effects, if any, would not influence biological interpretations. All samples were sequenced on two sequencing runs, with the first sequencing run containing the first set of replicates and the second containing the second and third set of replicates. PBS controls were included in every batch to ensure comparability, and transcriptomic profiles of PBS samples from different batches were computationally compared to verify batch-to-batch consistency (Extended Data Fig. 2a). In brief, Euclidean distances were calculated for each pair of PBS-treated cells of the same cell type based on the entire transcriptome to ensure that the within-batch distances and between-batch distances were comparable.

### Lymph node dissociation and cell sorting for scRNA-seq experiments

An optimized pipeline for viable cell recovery and more balanced cell-type representation was used to process lymph nodes for scRNA-seq. Lymph nodes were enzymatically digested using a protocol that maximizes the recovery of myeloid and stromal cells while maintaining high viability^40^. In brief, lymph nodes were placed in RPMI with collagenase IV, dispase and Dnase I at 37 °C, and cells were collected once they were detached. The cells were then immediately placed on ice and washed with PBS supplemented with 2 mM EDTA and 0.5% biotin-free BSA, then filtered through a 70 µm cell strainer. Cells were incubated with Fc blocking antibodies 4 °C, then with a biotinylated anti-CD3 and anti-CD19 antibody cocktail. Antibodies were used at a dilution of 1:100. Streptavidin microbeads were then added and the cells were magnetically sorted using MACS MS columns according to the manufacturer’s protocol (Miltenyi Biotec). After cell sorting, a small fraction of the CD3^+^ or CD19^+^ cells was pooled with CD3^–^CD19^–^ cells for more balanced representation of all cell types and proceeded immediately to scRNA-seq.

### scRNA-seq

Cell hashing was used to combine multiple samples into the same single-cell emulsion channel^41^. The mouse cells obtained from different stimulation conditions were stained with TotalSeq antibodies (BioLegend anti-mouse hashtags 1–8; used at 1:100 dilution), washed 5 times at 4 °C and pooled in PBS with 0.04% BSA according to the manufacturer’s protocol. Next, 55,000 cells were loaded onto a 10x Genomics Chromium instrument (10x Genomics) according to the manufacturer’s instructions. The scRNA-seq libraries were processed using a Chromium Single Cell 3′ Library & Gel Bead v3 kit (10x Genomics) with modifications for generating hashtag libraries^41^. Quality control for amplified cDNA libraries and final sequencing libraries was performed using a Bioanalyzer High Sensitivity DNA kit (Agilent). scRNA-seq and hashing libraries were normalized to 4 nM concentration and pooled. The pooled libraries were paired-end sequenced on a NovaSeq S4 platform targeting an average sequencing depth of 20,000 reads per cell for gene expression libraries, and on a NovaSeq S4 or SP platform targeting 5,000 reads per cell for hashtag libraries.

### scRNA-seq data pre-processing

The raw bcl sequencing data were processed using the CellRanger (v.3.0) Gene Expression pipeline (10x Genomics), including demultiplexing and alignment. Sequencing reads were aligned to the mm10 mouse reference genome, and transcriptomic count matrices were assembled. Hashtag library FASTQ files were processed using the CITE-seq-Count tool (v.1.4.3; github.com/Hoohm/CITE-seq-Count). Gene expression and hashtag were matched using the MULTIseqDemux function of the Seurat R package (v.4.1)^42^. Cells with multiple hashtags were considered multiplets (for example, doublets or triplets) and were excluded from further analysis. The Seurat R pipeline was used to perform quality control to include only cells with >500 genes, >1,000 unique molecular identifiers and <10% mitochondrial gene content. The expression matrix was globally scaled by normalizing the gene expression measurements by the total expression per cell. The resulting values were multiplied by a scale factor of 10,000 and natural log-transformed.

For the initial global analysis of all cells, the top 3,000 variable genes were selected for dimensionality reduction analysis. Principal component analysis (PCA) was then used to denoise and to find a lower-dimensional representation of the data. The top 75 principal components (PCs) were used for global clustering and for visualization using a *t*-SNE map^43^. Clusters were identified using the Louvain clustering algorithm. This step resulted in a total of 61 non-singleton global (level 1) clusters (Extended Data Fig. 1d). We removed potential multiplets by removing the cells with the top 2% gene counts in each cluster. As different cell types have variations in the numbers of genes detected on average, this step was done at the cluster level rather than for all data. For each level 1 cluster of cells, we then performed another round of clustering (level 2) to further verify the identity of each cluster and to remove potential doublets. This step resulted in a total of 183 global level 2 clusters. The cell-type identity of each level 2 cluster was assigned on the basis of the expression of 115 known marker genes (Supplementary Table 2). Clusters enriched for marker genes of multiple cell types were considered multiplets and removed. The top DEGs between each cell type and others are listed in Supplementary Table 2.

### Quantitative measures of reproducibility across animal replicates

A gene expression vector for each biological replicate was created for each cytokine stimulation condition in a given cell type by taking the difference between the average expression vector of cytokine-treated cells and the average expression vector of PBS-treated cells. Genes were included if they were significantly differentially expressed (FDR < 0.05 and absolute log_2_(FC) > 0.25) compared with PBS controls and were expressed in >10% of cytokine-treated cells for upregulated genes or >10% of PBS-treated cells for downregulated genes. *Rps*, *Rpl*, mitochondrial genes and unlabelled genes were excluded. Pairwise Pearson correlation coefficients were then calculated for these vectors (Extended Data Fig. 2b).

### Assessment of access to injected cytokines by each lymph node cell type

To determine whether the injected cytokines can be accessed by each cell type in the draining lymph nodes, we examined ISG expression levels in cells treated with IFNα1 or IFNβ (IFNα1/IFNβ) or PBS for each cell type. Type I interferon was chosen for this analysis given its strong induction of antiviral programmes in a wide range of cell types. A maximum of 100 cells were sampled from each condition. ISG scores were obtained by summing the normalized expressions of ISGs in each cell. These scores were then used to predict whether each cell was treated with IFNα1/IFNβ or PBS, and the accuracy of the prediction was represented as receiver operating characteristics curves (Extended Data Fig. 2c). The ISGs were obtained from the MSigDB hallmark gene set.

### Differential gene expression analysis

Analyses of DEGs were performed to identify marker genes for cell clusters or cytokine-responsive genes. Analyses of DEGs were performed between two groups of cells using the two-sided Wilcoxon rank-sum test on normalized gene expression values. The *P* values obtained from the tests were then adjusted (Bonferroni or FDR) to address multiple testing. Genes were considered DEGs in the cytokine signature if they had FDR < 0.05 and absolute log_2_(FC) > 0.25 between cytokine-treated and PBS-treated cells of the same cell type, were expressed in >10% of cytokine-treated cells for upregulated genes or >10% of PBS-treated cells for downregulated genes, and satisfied the FC threshold for at least two out of the three mice (to mitigate the influence of potential single-mouse outliers). *Rps*, *Rpl*, mitochondrial genes and unlabelled genes were excluded from the signatures. Cell-type-specific cytokine signatures are listed for all major cell types in Supplementary Table 3.

### Quantification of overall magnitude of transcriptomic responses to each cytokine in each cell type

We constructed a global reference map that quantified the overall gene expression changes induced by each cytokine in each cell type (Fig. 1d). This maps takes into account two metrics: the number of DEGs and the magnitude of change across the entire transcriptome. The number of DEGs is the total number of genes in each cytokine signature. The overall magnitude of cytokine-induced differential expression was computed as the Euclidean distance between the centroid vectors of cytokine-treated cells and PBS-treated cells. This value was normalized to a scale ranging from 0 (low) to 100 (high). To reduce the impact of outliers in the normalization process, winsorization was applied such that values above the 95th percentile were replaced with values at the 95th percentile before normalization. A maximum of 100 cells from each cytokine treatment condition were sampled for each cell type for the magnitude calculation. A distinct colour ramp was used for each cell type to emphasize that cell types have different properties (for example, different number of genes expressed on average) and were independently analysed. Cytokine–cell type combinations with five or more cells sampled were included in this analysis.

### Identification of GPs per cytokine treatment and per cell type

GP analysis was used to identify co-regulated genes for each cytokine treatment across cell types (Fig. 2c and Supplementary Fig. 1) or for each cell type across all treatment conditions (Extended Data Figs. 5i,j, 6i,j, 7i,j and 8i,j and Supplementary Figs. 2i,j, 3i,j, 4i,j, 5i,j, 6i,j, 7i,j, 8i,j, 9i,j, 10i,j and 11i,j). GPs were constructed using the non-negative matrix factorization (NMF) algorithm^44^ using the R package NMFN (v.2.0). We removed genes associated with tissue dissociation^45^ and the cell cycle, as well as mitochondrial genes, *Rps* and *Rpl*, unlabelled genes, globally overabundant genes and those expressed in fewer than ten cells.

In the cytokine-centric analysis, NMF was used to identify cell-type-specific GPs in response to each cytokine. Cells treated with the specified cytokines or PBS were used for NMF, with a maximum of 100 cells per cell type per condition. NMF was run separately for each cytokine in this analysis, except that IFNα1 and IFNβ were processed together and IL-1α and IL-1β were processed together in Fig. 2c and Supplementary Table 5. We identified 40 GPs per cytokine treatment, with some predominantly corresponding to cell-type identity and others predominantly to cellular responses to cytokine stimulation. To quantitatively identify GPs predominantly corresponding to cellular responses to cytokine stimulation, a two-sided Wilcoxon rank-sum test was used to identify GPs with weights that were significantly different between the cytokine-treated cells and PBS-treated cells. GPs showing significant upregulation in any cell types were displayed. The top 30 genes with the highest weights for each GP were used to identify enriched biological processes using clusterProfiler (v.4.2.1)^46^ on the Hallmark gene sets from the MSigDB database^47^. Genes highlighted in the text for biological significance satisfied two criteria: (1) they were among the top 30 highest-weighted genes for a significantly upregulated GP in response to cytokines; and (2) the genes individually also showed significant upregulation in response to cytokines in the DEG analysis of cytokine signatures.

In the cell-type-centric analysis, NMF was used to analyse GPs of each cell type across all cytokine treatment conditions to identify cytokines that induce similar cellular processes. We identified ten GPs for each cell type and visualized the relationship between GPs and subclusters for each cell type using heatmaps in Extended Data Fig. 5–8 and Supplementary Figs. 2–11. The top genes with the highest weights for each GP are shown in Extended Data Figs. 5j, 6j, 7j and 8j, Supplementary Figs. 2j, 3j, 4j, 5j, 6j, 7j, 8j, 9j, 10j and 11j and Supplementary Table 8.

### Analysis of secondary responses to induced IFNγ

The IFNγ-induced gene expression signature was used to infer the level of cellular responses to cytokine-induced IFNγ. The IFNγ signature score for each cell type was constructed by summing the expressions of significantly overexpressed genes in IFNγ-treated cells relative to PBS-treated cells (FDR-adjusted *P* < 0.001 and log_2_(FC) > 1) in the corresponding cell type. The log_2_(FC) of the signature scores and FDR-adjusted *P* value relative to PBS-treated cells were calculated for every cytokine treatment and shown in Extended Data Fig. 4e.

### Identification of cellular polarization states

To identify cellular polarization states induced by cytokines, subclustering was performed for cells of each cell type. For heterogeneous cell types (for example, macrophage, MigDC and γδ T cell), the most abundant homogeneous subset was analysed to identify cytokine-induced states instead of re-deriving cell subsets in the polarization state analysis. We used PCs to subcluster on the basis of discriminating genes, defined as genes with a large absolute log_2_(FC) (between 0.75 and 1.5 depending on cell type) in any cytokine-treated cells compared with PBS-treated cells. We removed genes associated with tissue dissociation^45^ and the cell cycle, as well as mitochondrial genes and *Rps* and *Rpl*. We then performed PCA and visualized the cells using UMAP^48^. The proportion of cells falling into each cluster was calculated for each cytokine or PBS control. Major polarization states were identified on basis of two criteria: (1) cell clusters with significantly (FDR-adjusted *P* < 0.01) more than the expected number of cytokine-stimulated cells using a hypergeometric test; and (2) manual verification of biological relevance of the highly expressed genes or GPs in the subcluster and cytokines inducing the changes. To find discriminating markers and biological functions of each state, we analysed DEGs and co-regulated GPs per state relative to all other cells for the cell type. DEGs were identified using the two-sided Wilcoxon rank-sum test between each polarization state and other cells of the same cell type. The significantly overexpressed genes with the largest log_2_(FC) are shown. The most strongly polarized states are summarized in Fig. 3. The complete landscape, including less-strongly polarized states, in each cell type can be found in Extended Data Figs. 5–8 and Supplementary Figs. 2–11. We compared the polarization states by calculating the pairwise Pearson correlation coefficients between the gene expression profiles of each polarization state after subtracting the profiles of PBS-treated cells of the same cell type to remove cell-type-specific gene expression. These results are displayed in Extended Data Figs. 5b, 6b, 7b and 8b and Supplementary Figs. 2b, 3b, 4b, 5b, 6b, 7b, 8b, 9b, 10b and 11b.

We assigned a unique identifier to each polarization state using the following convention: ‘<cell type abbreviation>-<lower case letters>’. When applicable, the letters a–d were reserved for type I interferon, type II interferon, IL-1α and IL-1β, and TNF, respectively, which are cytokines that induce polarization states across a large number of cell types.

### Comparative global view of polarization states across immune cell types

To gain a global view of the 66 polarization states across immune cell types defined in Fig. 3, we used Jaccard similarity index to evaluate similarity between each pair of cell states (Extended Data Fig. 9). The gene expression profile of each polarization state was compared with PBS-treated cells of the same cell type to remove cell-type-specific gene expression. The genes with an absolute log_2_(FC) > 0.5 compared with PBS-treated cells were used to compute the Jaccard similarity score. Upregulated and downregulated genes were separately calculated. The rows and columns were hierarchically clustered using the average-linkage method on the Euclidean distances to identify groups of similar polarization states. To visualize unique polarization states with low similarity to other states, the same results were illustrated using a force-directed network, with a higher circle size indicating a more unique state, which was calculated on the basis of the inverse of mean Jaccard similarity value with other states.

### Pathway enrichment analysis for NK cells

To identify biological processes enriched for the IL-18-treated NK cells, a pre-ranked gene list was computed by subtracting average gene expression values of PBS-treated NK cells from those in IL-18-treated NK cells. Gene set enrichment analysis was performed using clusterProfiler (v.4.2.1) on the gene ontology biological processes gene sets. Gene sets with a FDR-adjusted *P* < 0.1 are shown. As a comparison, representative cytokines from other NK cell polarization states were analysed using the same method.

### Cytokine and cytokine–receptor gene expression maps

A map of cell-type-specific production of cytokines was derived from our dataset. Cytokine genes expressed in at least 50 cells were included in the cytokine expression heatmap. The cells were obtained from all conditions (PBS or cytokine treated) to provide a map of cytokine expression under all unstimulated or cytokine stimulation conditions (that is, to account for induced expression). The gene expression level was then normalized relative to the cell type with the maximum expression level (whereby the maximum level is capped at 1 expression unit before normalization) to account for the variation in the number of transcripts produced or detected for each cytokine. A cytokine was considered expressed in a cell type if more than 0.1 normalized expression units were detected.

The cytokine–receptor expression map was constructed using the same approach. This included signalling receptors, decoy receptors and receptors that form complexes with cytokines. A list of genes encoding known functional receptors for the 86 studied cytokines are listed in Supplementary Table 1. The cytokine expression map and the cytokine–receptor expression map are shown in Fig. 4a, Extended Data Fig. 10c and Supplementary Table 9.

### Cell–cell interactome network construction

A cell–cell interactome network was constructed to chart available cytokine-mediated cell–cell communication channels. The network was constructed such that the source and sink nodes are cell types and intermediate nodes are cytokines. The paths between source cell-type nodes and sink cell-type nodes through cytokine nodes were established on the basis of the detectability of the cytokine mRNA in the cell population (normalized expression > 0.1) and the responsiveness of the cell type to the cytokine (more than ten DEGs in the corresponding cytokine signature). For heteromeric cytokines or cytokine complexes composed of two subunits (IL-12, IL-23, IL-27, LTα1/β2 and LTα2/β1), the cytokine is shown as expressed and is annotated with an asterisk if the genes encoding at least one subunit are expressed as there is evidence of extracellular assembly of some components into functional cytokines under healthy or pathological conditions^49^. The network was plotted separately for each source node for ease of interpretability.

To construct the ligand–receptor interactome, we identified functional cognate receptors for each cytokine from the literature, which is listed in Supplementary Table 1. For a receptor to be considered expressed in a cell type, the normalized expression value of the receptor gene needed to be greater than a cut-off threshold (default of 0.1 expression unit). For heteromeric receptors, all components needed to be expressed for the receptor to be considered expressed. For cytokines with more than one functional receptor, the receptor was considered expressed if any functional receptors are expressed. We then connected the cytokines with the cell types expressing the cognate receptors. The cytokine production portion of the interactome is the same as the one in the ligand–response interactome. The ligand–response and ligand–receptor networks were then compared to generate the cell–cell communication paths that are common or different between these two approaches.

### IREA for cytokine response analysis

We offer two types of IREA analysis options to assess cytokine responses in a user’s data depending on the input, which can be a gene set or a gene expression matrix. The cell type in the user data is specified by the user. User data are then compared with the transcriptional cytokine responses of the same cell type from the Immune Dictionary using the following methods:

1. For the gene set input, we first find gene set scores by summing the normalized expression value of all genes in the gene set in each of the cytokine-treated cells or PBS-treated cells. Statistical significance is assessed using a two-sided Wilcoxon rank-sum test between gene set scores on cytokine-treated cells and gene set scores on PBS-treated cells, and an FDR correction is applied to all cytokine calculations. Enrichment can also be calculated using the hypergeometric test on significant DEGs (FDR < 0.01 between cytokine-treated cells and PBS-treated cells), a method commonly used in pathway analyses.
2. For the gene expression matrix input, the expression matrices are first normalized such that the total expression per cell sums to 10,000 units; the expression is then log-transformed. Genes giving significant contribution to the enrichment score, with the default being those having an average of more than 0.25 expression values, were included. Next, the projection score is calculated by finding the cosine similarity score between user input and cytokine-treated or PBS-treated cells. Statistical significance is assessed using a two-sided Wilcoxon rank-sum test between projection scores on cytokine-treated cells and projection scores on PBS-treated cells, and an FDR correction is applied to all cytokine calculations. The effect size is the mean difference between projection scores on cytokine-treated cells and on PBS-treated cells. The effect size and FDR-adjusted *P* value for each of the 86 cytokines can then be visualized using a compass plot shown in Fig. 5d. Conceptually, this method takes into consideration the direction and magnitude of expression of each gene. That is, a strongly upregulated gene in both the user dataset and Immune Dictionary reference dataset is given a high weight that increases the overall likelihood of enrichment; a strongly upregulated gene in one dataset but not the other is given lower weight; and a gene that is upregulated in one dataset but downregulated in the other is given negative weight that decreases the overall likelihood of enrichment. The genes contributing the highest weights to the enrichment can be visualized using a diverging bar plot shown in Extended Data Fig. 12b.

### IREA for cellular polarization analysis

IREA polarization analysis implements the same statistical test as the IREA cytokine response analysis. In IREA polarization, user data are compared with the polarization state gene expression profiles. A polarized radar plot is shown if at least one cellular polarization state is significantly enriched (FDR-adjusted *P* < 0.05). If no state is significantly enriched, the radar plot shows an enrichment score of 0 for every state, which signifies that the input cells are unpolarized. The enrichment score is normalized to be between 0 and 1 on the radar plot.

### IREA for cell–cell communication network construction

We constructed models of cell–cell communication networks by taking into account cytokine production and cytokine response. Cytokine production was obtained by examining the transcripts mapped to each of the 86 cytokines. The cytokine response was assessed using IREA, and cytokines with IREA output of FDR < 0.01 were included. For heteromeric cytokines or cytokine complexes composed of two subunits, the cytokine was displayed as expressed if at least one subunit is expressed as there is evidence of extracellular assembly of some components into functional cytokines^49^ (same method as for the cell–cell interactome). Cytokine networks can be visualized as shown in Fig. 5e and Extended Data Fig. 12c.

### IREA analysis of mouse tumour scRNA-seq data

The scRNA-seq data were downloaded as 10x Genomics data files^36^. Data were processed using the same approach as described above but with a minor modification, whereby 40 PCs were used for downstream analysis. Cell types were annotated as shown in the publication^36^. The IREA analysis was done between anti-PD-1 treatment and controls for each cell type using the transcriptome-wide approach with default parameters. A receptor expression threshold of 0.05 was applied to produce the data in the receptor ring in the cytokine enrichment plot.

### IREA analysis of human COVID-19 blood scRNA-seq data

The scRNA-seq data were downloaded as a Seurat object from the human COVID-19 blood study^39^. Cluster annotations were used as defined in the Seurat object. IREA analysis was performed using data from ventilated patients with COVID-19 and compared with healthy individuals for each cell type using the transcriptome-wide approach with default parameters and species specified as human. IREA implements mouse and human homologue gene conversion using the most recent release of the National Center for Biotechnology Information HomoloGene database (release 68). A receptor expression threshold of 0.05 was applied to produce the data in the receptor ring in the compass plot.

### Statistical analysis

The statistical tests used are described for each analysis in the corresponding text. Two-sided statistical tests were used unless otherwise specified. FDR or Bonferroni adjustments were made for the analyses for which multiple hypothesis testing applies.

### Reporting summary

Further information on research design is available in the Nature Portfolio Reporting Summary linked to this article.

## Online content

Any methods, additional references, Nature Portfolio reporting summaries, source data, extended data, supplementary information, acknowledgements, peer review information; details of author contributions and competing interests; and statements of data and code availability are available at 10.1038/s41586-023-06816-9.

### Supplementary information

Supplementary Information Supplementary Figs. 1–14.

Reporting Summary

Supplementary Table 1 Cytokines studied, alternative names, corresponding gene symbols, known cognate receptors, and selected prior in vivo studies. Table 1: Listing of 86 cytokines studied and their family, alternative names, mouse and human gene symbols, and gene symbols for the corresponding known mouse and human cytokine receptors. Table 2: Listing of 116 prior publications that evaluated the *in vivo* effects of studied cytokines and doses used.

Supplementary Table 2 Marker genes for each global cluster and each cell type in the scRNA-seq data. Table 1: Listing of 115 marker gene expressions across 183 global level-2 clusters. Percentage of cells expressing each gene, scaled average expression, and annotated cell type identity are shown. Table 2: Listing of top DEGs between each level-2 cluster and all other clusters. Shown for log_2_FC, percentage of cells expressing the gene in the cluster vs. in all other clusters, and two-sided Wilcoxon rank-sum test *P*-value and Bonferroni-adjusted *P*-value for the differential gene expression test. Table 3: Listing of top DEGs between each annotated cell type and all other cell types.

Supplementary Table 3 Cytokine signatures: DEGs in response to each cytokine treatment in each cell type. Listing of cytokine treatments, DEGs, log_2_ fold change between cytokine- and PBS-treatments, FDR-adjusted *P*-values from two-sided Wilcoxon rank-sum test for the differential gene expression analysis, and percentages of cells expressing the gene in cytokine-treated cells and in PBS-treated cells. Each tab is a separate cell type. These cytokine signatures are summarized in Fig. 1d.

Supplementary Table 4 Cytokine-centric analysis: Listing of gene symbols, fold change, percentage of cells expressing the gene in the cytokine treatment and in PBS treatment. Listing of gene symbols, fold change, percentage of cells expressing the gene in the cluster and in all other clusters, and two-sided Wilcoxon rank-sum test *P*-value and FDR-adjusted *P*-value for the differential gene expression test in Fig. 2b. The analysis was performed on cytokine-treated cells vs. PBS-treated cells of the same cell type, with a maximum of 100 cells sampled for cytokine treatment and 300 cells for PBS treatment for each cell type to ensure comparability across cell types.

Supplementary Table 5 Cytokine-centric analysis: Gene programs in NMF analyses. Listing of 30 genes with the top weights in each gene program for each cytokine as shown in Fig. 2c and Supplementary Fig. 1. NMF is performed on all main cell types for each cytokine independently.

Supplementary Table 6 Cell-type-centric analysis: Subcluster markers for each immune cell type. Listing of top DEGs (log_2_FC > 0.25) between each subcluster and all other subclusters in each cell type (i.e., full listing of DEGs for panel e in Extended Data Figs. 5-8 and Supplementary Figs. 2-11). Shown for log_2_FC, percentage of cells expressing the gene in the subcluster vs. in all other subclusters, and two-sided Wilcoxon rank-sum test *P*-value and Bonferroni-adjusted *P*-value for the differential gene expression test. Each tab is a separate cell type.

Supplementary Table 7 Cell-type-centric analysis: Markers of polarization states for each immune cell type. Listing of top DEGs (log_2_FC > 0.25 and Bonferroni-adjusted *P*-value < 0.05) between cells in each polarization state (Fig. 3) and all other cells of the same cell type. Shown for log_2_FC, percentage of cells expressing the gene in the state vs. in all other cells, and two-sided Wilcoxon rank-sum test *P*-value and Bonferroni-adjusted *P*-value for the differential gene expression test. Each tab is a separate cell type.

Supplementary Table 8 Cell-type-centric analysis: Gene program analysis for each immune cell type. Listing of 30 top-weighted genes in each gene program for each cytokine as shown in panel j in Extended Data Figs. 5-8 and Supplementary Figs. 2-11. NMF is performed on all cytokine treatments in each cell type. Each tab is a separate cell type.

Supplementary Table 9 Expression maps of cytokine and cytokine receptor genes in each cell type. Table 1: Row-normalized expression levels of genes encoding the 86 studied cytokines, as shown in Fig. 4a. Table 2: Unnormalized expression levels of genes encoding the 86 studied cytokines. Table 3: Row-normalized expression levels of genes encoding known receptors for the 86 studied cytokines, as shown in Extended Data Fig. 10c. Table 4: Unnormalized expression levels of genes encoding known receptors for the 86 studied cytokines.

Supplementary Table 10 A draft cytokine-mediated cell-cell interactome network. Table 1: Connections between cytokines and target cells using the ligand-response approach. Table 2: Connections between cytokines and target cells using the ligand-receptor approach. Table 3: Connections found in the ligand-response network that are not found in the ligand-receptor network. Table 4: Connections found in the ligand-receptor network that are not found in the ligand-response network. Table 5: Connections found in both ligand-response and ligand-receptor networks.

### Source data

Source Data Fig. 1

Source Data Fig. 2

Source Data Fig. 3

Source Data Fig. 4

Source Data Fig. 5

Source Data Extended Data Fig. 1

Source Data Extended Data Fig. 2

Source Data Extended Data Fig. 3

Source Data Extended Data Fig. 4

Source Data Extended Data Fig. 5

Source Data Extended Data Fig. 6

Source Data Extended Data Fig. 7

Source Data Extended Data Fig. 8

Source Data Extended Data Fig. 9

Source Data Extended Data Fig. 10

Source Data Extended Data Fig. 11

Source Data Extended Data Fig. 12

## Data Availability

The Immune Dictionary interactive web portal can be accessed at www.immune-dictionary.org, where the single-cell transcriptomic data generated in this study are made publicly available through a user-friendly interface. Raw fastq files of the data are available from the Gene Expression Omnibus (GEO) database under accession number GSE202186. Source tables are included for every figure and extended data figure at appropriate locations in the article. In addition, the following publicly available datasets were used: the MSigDB database was used for annotating biological processes and can be accessed at www.gsea-msigdb.org/gsea/msigdb; mouse and human homologue gene conversion was based on the National Center for Biotechnology Information HomoloGene database (release 68); the mouse tumour dataset is available from the GEO under accession number GSE119352; and the human COVID-19 dataset is available at www.covid19cellatlas.org. Source data are provided with this paper.

## Code availability

IREA is available at www.immune-dictionary.org.

## References

[1] Arai KI (1990). Cytokines: coordinators of immune and inflammatory responses. Annu. Rev. Biochem..

[2] Briukhovetska D (2021). Interleukins in cancer: from biology to therapy. Nat. Rev. Cancer.

[3] Ye, Q., Wang, B. & Mao, J. The pathogenesis and treatment of the ‘cytokine storm’ in COVID-19. *J. Infect.*10.1016/j.jinf.2020.03.037 (2020). 10.1016/j.jinf.2020.03.037 PMC7194613 32283152

[4] Sims JE, Smith DE (2010). The IL-1 family: regulators of immunity. Nat. Rev. Immunol..

[5] Rochman Y, Spolski R, Leonard WJ (2009). New insights into the regulation of T cells by γc family cytokines. Nat. Rev. Immunol..

[6] Pestka S, Krause CD, Walter MR (2004). Interferons, interferon-like cytokines, and their receptors. Immunol. Rev..

[7] Jones SA, Jenkins BJ (2018). Recent insights into targeting the IL-6 cytokine family in inflammatory diseases and cancer. Nat. Rev. Immunol..

[8] Korn T, Bettelli E, Oukka M, Kuchroo VK (2009). IL-17 and Th17 cells. Annu. Rev. Immunol..

[9] Locksley RM, Killeen N, Lenardo MJ (2001). The TNF and TNF receptor superfamilies: integrating mammalian biology. Cell.

[10] Akdis M (2016). Interleukins (from IL-1 to IL-38), interferons, transforming growth factor β, and TNF-α: receptors, functions, and roles in diseases. J. Allergy Clin. Immunol..

[11] Urrutia A (2016). Standardized whole-blood transcriptional profiling enables the deconvolution of complex induced immune responses. Cell Rep..

[12] Subramanian A (2017). A next generation connectivity map: L1000 platform and the first 1,000,000 profiles. Cell.

[13] Xue J (2014). Transcriptome-based network analysis reveals a spectrum model of human macrophage activation. Immunity.

[14] Saxton RA, Glassman CR, Garcia KC (2023). Emerging principles of cytokine pharmacology and therapeutics. Nat. Rev. Drug Discov..

[15] Mostafavi S (2016). Parsing the interferon transcriptional network and Its disease associations. Cell.

[16] Amit I (2009). Unbiased reconstruction of a mammalian transcriptional network mediating pathogen responses. Science.

[17] Merad M, Ginhoux F, Collin M (2008). Origin, homeostasis and function of Langerhans cells and other langerin-expressing dendritic cells. Nat. Rev. Immunol..

[18] Gardner JM (2008). Deletional tolerance mediated by extrathymic Aire-expressing cells. Science.

[19] Dinarello CA (2018). Overview of the IL-1 family in innate inflammation and acquired immunity. Immunol. Rev..

[20] Ozaki K, Leonard WJ (2002). Cytokine and cytokine receptor pleiotropy and redundancy. J. Biol. Chem..

[21] Murray PJ (2017). Macrophage polarization. Annu. Rev. Physiol..

[22] Martinez FO, Gordon S (2014). The M1 and M2 paradigm of macrophage activation: time for reassessment. F1000Prime Rep..

[23] Crinier A (2018). High-dimensional single-cell analysis identifies organ-specific signatures and conserved NK cell subsets in humans and mice. Immunity.

[24] Lavin Y (2014). Tissue-resident macrophage enhancer landscapes are shaped by the local microenvironment. Cell.

[25] Freud AG, Mundy-Bosse BL, Yu J, Caligiuri MA (2017). The broad spectrum of human natural killer cell diversity. Immunity.

[26] Zhou T (2020). IL-18BP is a secreted immune checkpoint and barrier to IL-18 immunotherapy. Nature.

[27] Yasuda K, Nakanishi K, Tsutsui H (2019). Interleukin-18 in health and disease. Int. J. Mol. Sci..

[28] Park K (2016). The transcription factor NR4A3 controls CD103^+^ dendritic cell migration. J. Clin. Invest..

[29] Krishnamurty AT, Turley SJ (2020). Lymph node stromal cells: cartographers of the immune system. Nat. Immunol..

[30] Stubbington MJT, Rozenblatt-Rosen O, Regev A, Teichmann SA (2017). Single-cell transcriptomics to explore the immune system in health and disease. Science.

[31] Papalexi E, Satija R (2018). Single-cell RNA sequencing to explore immune cell heterogeneity. Nat. Rev. Immunol..

[32] Jiang P (2021). Systematic investigation of cytokine signaling activity at the tissue and single-cell levels. Nat. Methods.

[33] Browaeys R, Saelens W, Saeys Y (2020). NicheNet: modeling intercellular communication by linking ligands to target genes. Nat. Methods.

[34] Vento-Tormo R (2018). Single-cell reconstruction of the early maternal–fetal interface in humans. Nature.

[35] Ramilowski JA (2015). A draft network of ligand–receptor-mediated multicellular signalling in human. Nat. Commun..

[36] Gubin MM (2018). High-dimensional analysis delineates myeloid and lymphoid compartment remodeling during successful immune-checkpoint cancer therapy. Cell.

[37] Garris CS (2018). Successful anti-PD-1 cancer immunotherapy requires T cell–dendritic cell crosstalk involving the cytokines IFN-γ and IL-12. Immunity.

[38] Mariathasan S (2018). TGFβ attenuates tumour response to PD-L1 blockade by contributing to exclusion of T cells. Nature.

[39] Wilk AJ (2020). A single-cell atlas of the peripheral immune response in patients with severe COVID-19. Nat. Med..

[40] Fletcher, A. L. et al. Reproducible isolation of lymph node stromal cells reveals site-dependent differences in fibroblastic reticular cells. *Front. Immunol.*10.3389/fimmu.2011.00035 (2011). 10.3389/fimmu.2011.00035 PMC3342056 22566825

[41] Stoeckius M (2018). Cell hashing with barcoded antibodies enables multiplexing and doublet detection for single cell genomics. Genome Biol..

[42] Hao Y (2021). Integrated analysis of multimodal single-cell data. Cell.

[43] van der Maaten L, Hinton G (2008). Visualizing data using t-SNE. J. Mach. Learn. Res..

[44] Lee, D. D. & Sebastian Seung, H. Learning the parts of objects by non-negative matrix factorization. *Nature*10.1038/44565 (1999). 10.1038/44565 10548103

[45] van den Brink SC (2017). Single-cell sequencing reveals dissociation-induced gene expression in tissue subpopulations. Nat. Methods.

[46] Wu T (2021). clusterProfiler 4.0: a universal enrichment tool for interpreting omics data. Innovation.

[47] Subramanian A (2005). Gene set enrichment analysis: a knowledge-based approach for interpreting genome-wide expression profiles. Proc. Natl Acad. Sci. USA.

[48] Becht, E. et al. Dimensionality reduction for visualizing single-cell data using UMAP. *Nat. Biotechnol.*10.1038/nbt.4314 (2018). 10.1038/nbt.4314 30531897

[49] Abdi K (2014). Free IL-12p40 monomer Is a polyfunctional adaptor for generating novel IL-12–like heterodimers extracellularly. J. Immunol..

