# Supplementary material for: Dictionary of immune responses to cytokines at single-cell resolution
Source: Nature. 2023 Dec 6;625(7994):377–84. doi: 10.1038/s41586-023-06816-9 (PMC10781646; doi:10.1038/s41586-023-06816-9)
Supplement: Supplementary file 1 — Supplementary Figs. 1–14. [file 41586_2023_6816_MOESM1_ESM.pdf]

nature portfolio

https://doi.org/10.1038/s41586-023-06816-9

Supplementary information

Dictionary of immune responses to cytokines at single-cell resolution

In the format provided by the authors and unedited

Supplementary Guide

Dictionary of immune responses to cytokines at single-cell resolution

Ang Cui[1,2,12,*], Teddy Huang[3], Shuqiang Li[2,3], Aileen Ma[2,4], Jorge L. Pérez[2,4], Chris Sander[2,5,6], Derin B. Keskin[3,7], Catherine J. Wu[2,7,8], Ernest Fraenkel[2,9], Nir Hacohen[2,10,11,*]

[1]Harvard-MIT Division of Health Sciences and Technology, Massachusetts Institute of Technology, Cambridge, MA, USA
[2]Broad Institute of MIT and Harvard, Cambridge, MA, USA
[3]Translational Immunogenomics Lab, Dana-Farber Cancer Institute, Boston, MA, USA
[4]Department of Electrical Engineering and Computer Science, Massachusetts Institute of Technology, Cambridge, MA, USA
[5]Department of Cell Biology, Harvard Medical School, Boston, MA, USA
[6]cBio Center, Department of Data Science, Dana-Farber Cancer Institute, Boston, MA, USA
[7]Department of Medical Oncology, Dana-Farber Cancer Institute, Boston, MA, USA
[8]Department of Medicine, Brigham and Women's Hospital, Harvard Medical School, Boston, MA, USA
[9]Department of Biological Engineering, Massachusetts Institute of Technology, Cambridge, MA, USA
[10]Department of Medicine, Massachusetts General Hospital, Harvard Medical School, Boston, MA, USA
[11]Center for Cancer Research, Massachusetts General Hospital, Boston, MA, USA
[12]Present address: Faculty of Medicine, Harvard University, Boston, MA, USA

*Corresponding authors:
Ang Cui: ang_cui@g.harvard.edu
Nir Hacohen: nhacohen@mgh.harvard.edu

Table of Contents

***Supplementary Figures*......3**

Supplementary Fig. 1 | Gene programs (GPs) upregulated by IFN-γ, IL-18, IL-33, IL-36α, IL-2, IL-4, IL-7, IL-15, IL-3, GM-CSF, and TNF-α across cell types. 4

Supplementary Fig. 2 | CD4+ T cell responses to cytokines: polarization states, subclusters, marker gene expression, and gene programs. 6

Supplementary Fig. 3 | CD8+ T cell responses to cytokines: polarization states, subclusters, marker gene expression, and gene programs. 8

Supplementary Fig. 4 | γδ T cell responses to cytokines: polarization states, subclusters, marker gene expression, and gene programs. 10

Supplementary Fig. 5 | Treg responses to cytokines: polarization states, subclusters, marker gene expression, and gene programs. 12

Supplementary Fig. 6 | pDC responses to cytokines: polarization states, subclusters, marker gene expression, and gene programs. 14

Supplementary Fig. 7 | cDC2 responses to cytokines: polarization states, subclusters, marker gene expression, and gene programs. 16

Supplementary Fig. 8 | MigDC responses to cytokines: polarization states, subclusters, marker gene expression, and gene programs. 18

Supplementary Fig. 9 | Langerhans cell responses to cytokines: polarization states, subclusters, marker gene expression, and gene programs. 20

Supplementary Fig. 10 | Monocyte responses to cytokines: polarization states, subclusters, marker gene expression, and gene programs. 22

Supplementary Fig. 11 | Neutrophil responses to cytokines: polarization states, subclusters, marker gene expression, and gene programs. 24

Supplementary Fig. 12 | Further characterization of IL-18 induced NK-f polarization state and IL-1α/β induced NK-c polarization state in NK cells. 26

Supplementary Fig. 13 | Cell type properties in the cell-cell interactome. 28

Supplementary Fig. 14 | Additional IREA software output on human COVID-19 blood samples... 29

Supplementary Figures

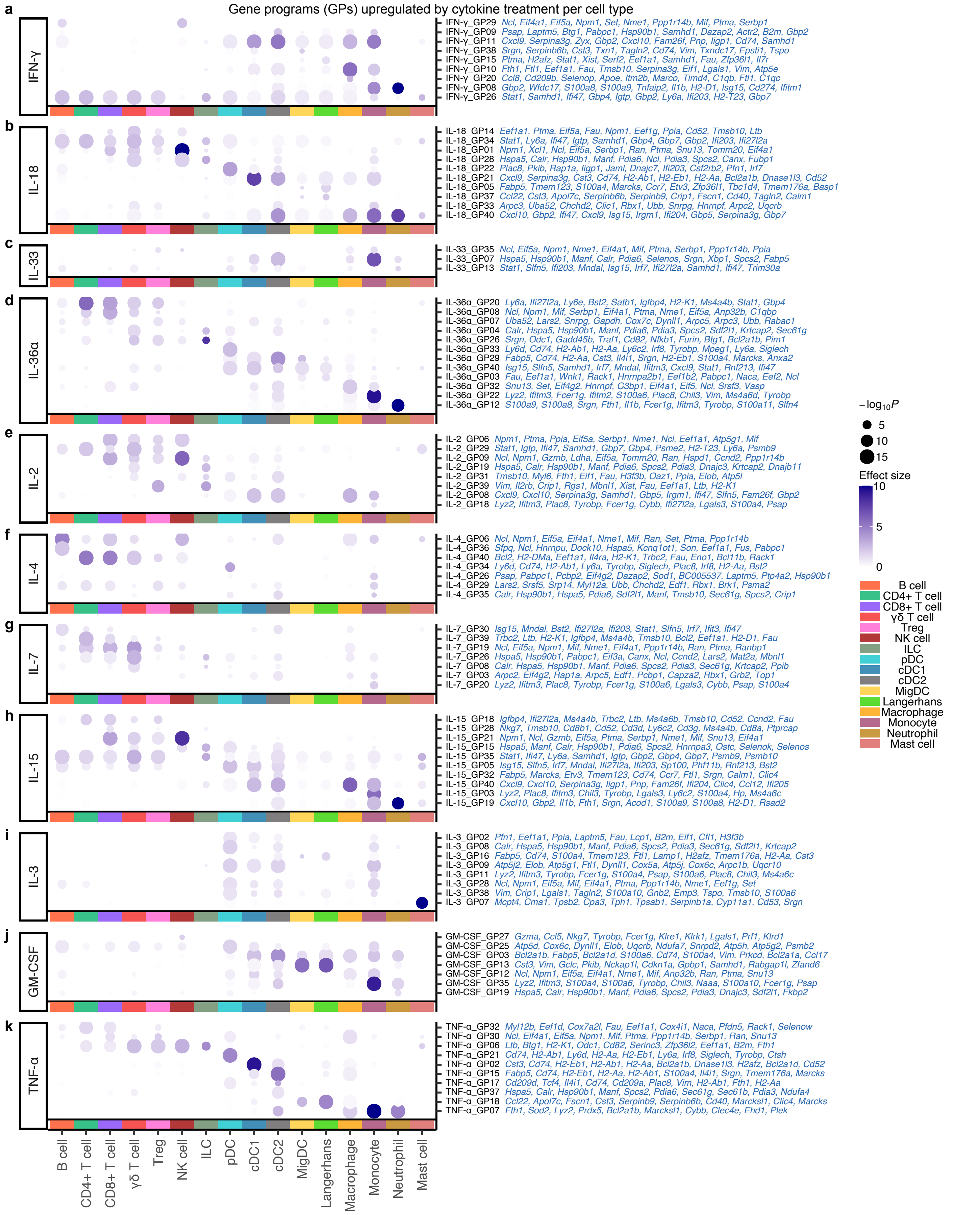

Supplementary Fig. 1 | Gene programs (GPs) upregulated by IFN-γ, IL-18, IL-33, IL-36α, IL-2, IL-4, IL-7, IL-15, IL-3, GM-CSF, and TNF-α across cell types.

See next page for caption.

Supplementary Fig. 1 | Gene programs (GPs) upregulated by IFN-γ, IL-18, IL-33, IL-36α, IL-2, IL-4, IL-7, IL-15, IL-3, GM-CSF, and TNF-α across cell types.

a-k, Upregulated GPs following cytokine treatment with respect to PBS controls for **a,** IFN-γ, **b,** IL-18, **c,** IL-33, **d,** IL-36α, **e,** IL-2, **f,** IL-4, **g,** IL-7, **h,** IL-15, **i,** IL-3, **j,** GM-CSF, and **k,** TNF-α. GPs that are significantly upregulated between cytokine and PBS treatment (FDR < 0.01 and effect size > 1) in any cell type are shown in the figure. Significant GPs (FDR < 0.05 and effect size > 0) for each cell type are represented as circles, with circle size indicating statistical significance and color representing effect size (capped at 10). The top-weighted genes in each upregulated GP, which are co-expressed genes that may be related to cell type identity or cellular response to cytokines, are shown on the right in blue.

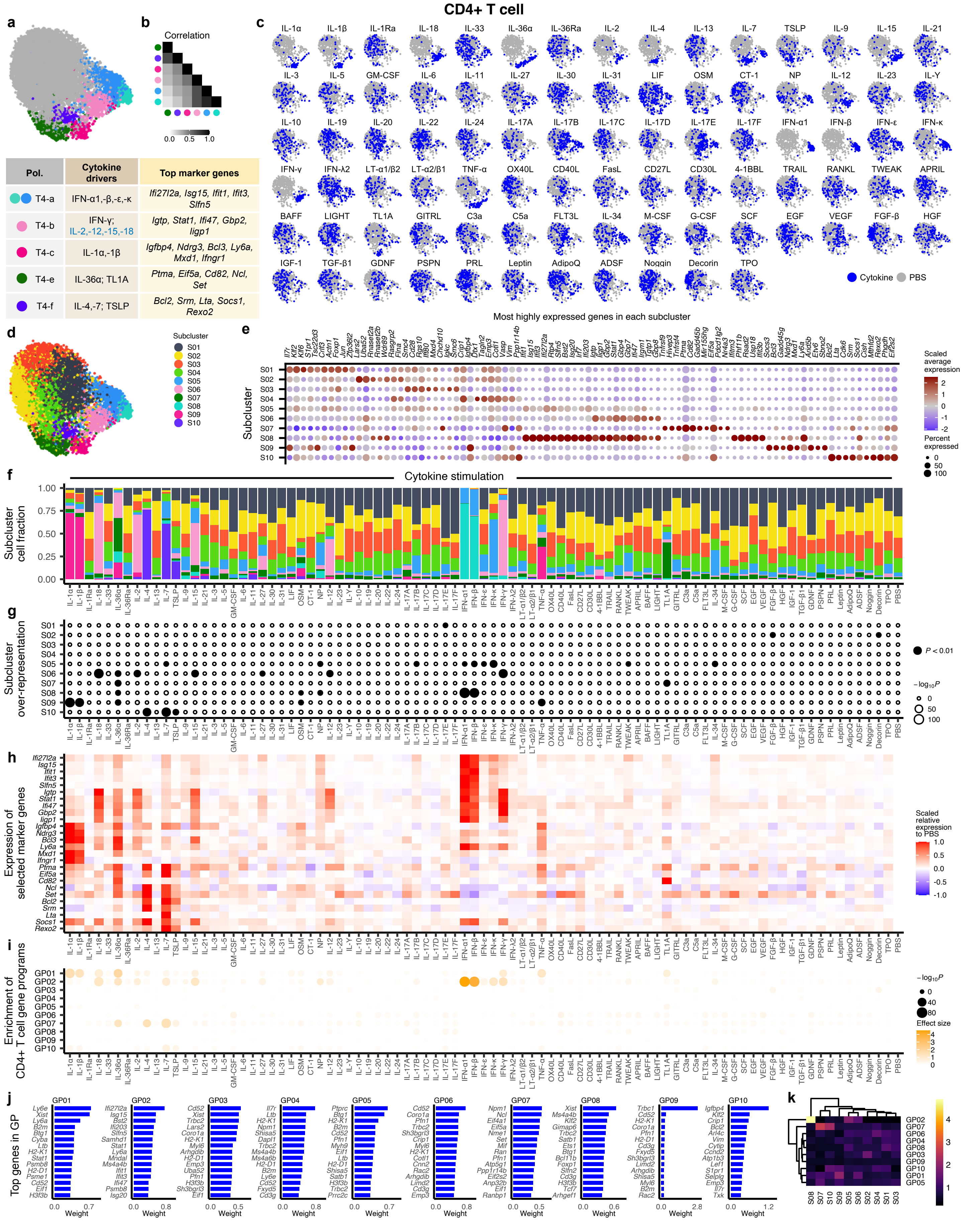

Supplementary Fig. 2 | CD4+ T cell responses to cytokines: polarization states, subclusters, marker gene expression, and gene programs. See next page for caption.

Supplementary Fig. 2 | CD4+ T cell responses to cytokines: polarization states, subclusters, marker gene expression, and gene programs.

a, Top, UMAP visualization of CD4+ T cells for all cytokines, colored by polarization states; bottom, table with cell type polarization states (left column), single cytokine drivers (middle column), and top marker genes (right column); reproduced from **Fig. 3** for ease of reference. **b**, Pairwise Pearson correlation coefficients between polarization states. **c**, UMAP visualization of CD4+ T cells shown independently for each cytokine treatment, colored by cytokine treatment (blue) or PBS treatment control (gray). **d**, UMAP visualization of CD4+ T cells for all cytokine or PBS treatment; cells colored for CD4+ T cell Louvain subclusters. **e**, Top overexpressed genes in each Louvain subcluster in **d**; color, column-scaled average expression; size of circle, percentage of cells in the subcluster expressing each gene. **f-i**, CD4+ T cell responses to each cytokine stimulation. **f**, Fraction of cells per subcluster in each cytokine treatment. Colors represent subclusters defined in **d**. **g**, Enrichment of each subcluster in each cytokine treatment; size of circle, Bonferroni-adjusted *P*-value of hypergeometric test relative to PBS; black fills, $P < 0.01$. **h**, Row-normalized relative expression of representative marker genes of each polarization state in cytokine-treated vs. PBS-treated cells. **i**, Enrichment of CD4+ T cell gene programs obtained from NMF analysis of all CD4+ T cells in cytokine-treated cells relative to PBS-treated cells; size of circle, FDR-adjusted *P*-value from two-sided Wilcoxon rank-sum test; shade, effect size representing the mean difference in gene program weight. **j**, Top weighted genes in each gene program in **i**. **k**, Average gene program weight in each subcluster. Rows and columns were hierarchically clustered using the complete-linkage method on Euclidean distances.

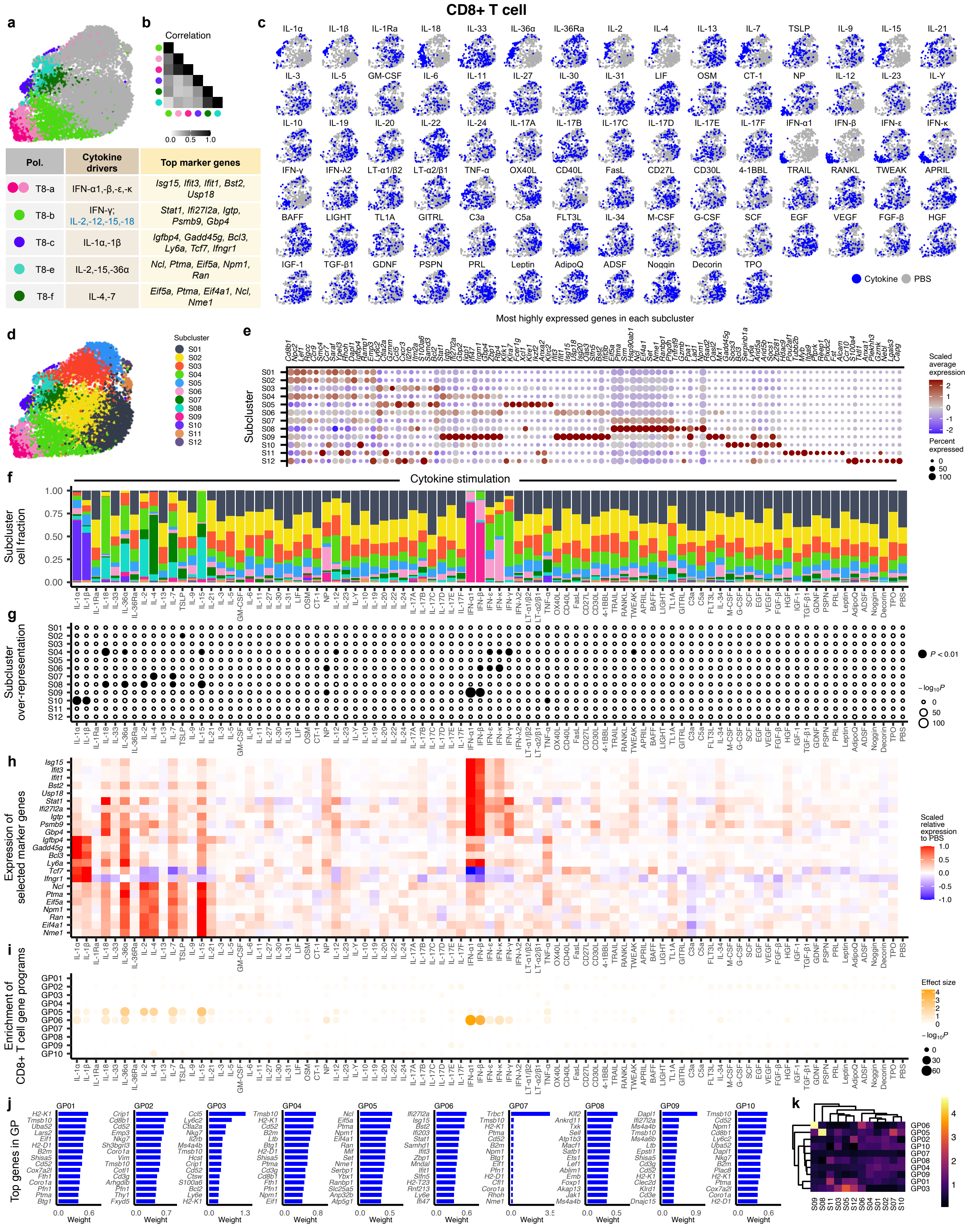

Supplementary Fig. 3 | CD8+ T cell responses to cytokines: polarization states, subclusters, marker gene expression, and gene programs.
See next page for caption.

Supplementary Fig. 3 | CD8+ T cell responses to cytokines: polarization states, subclusters, marker gene expression, and gene programs.

a, Top, UMAP visualization of CD8+ T cells for all cytokines, colored by polarization states; bottom, table with cell type polarization states (left column), single cytokine drivers (middle column), and top marker genes (right column); reproduced from **Fig. 3** for ease of reference. **b**, Pairwise Pearson correlation coefficients between polarization states. **c**, UMAP visualization of CD8+ T cells shown independently for each cytokine treatment, colored by cytokine treatment (blue) or PBS treatment control (gray). **d**, UMAP visualization of CD8+ T cells for all cytokine or PBS treatment; cells colored for CD8+ T cell Louvain subclusters. **e**, Top overexpressed genes in each Louvain subcluster in **d**; color, column-scaled average expression; size of circle, percentage of cells in the subcluster expressing each gene. **f-i**, CD8+ T cell responses to each cytokine stimulation. **f**, Fraction of cells per subcluster in each cytokine treatment. Colors represent subclusters defined in **d**. **g**, Enrichment of each subcluster in each cytokine treatment; size of circle, Bonferroni-adjusted *P*-value of hypergeometric test relative to PBS; black fills, $P < 0.01$. **h**, Row-normalized relative expression of representative marker genes of each polarization state in cytokine-treated vs. PBS-treated cells. **i**, Enrichment of CD8+ T cell gene programs obtained from NMF analysis of all CD8+ T cells in cytokine-treated cells relative to PBS-treated cells; size of circle, FDR-adjusted *P*-value from two-sided Wilcoxon rank-sum test; shade, effect size representing the mean difference in gene program weight. **j**, Top weighted genes in each gene program in **i**. **k**, Average gene program weight in each subcluster. Rows and columns were hierarchically clustered using the complete-linkage method on Euclidean distances.

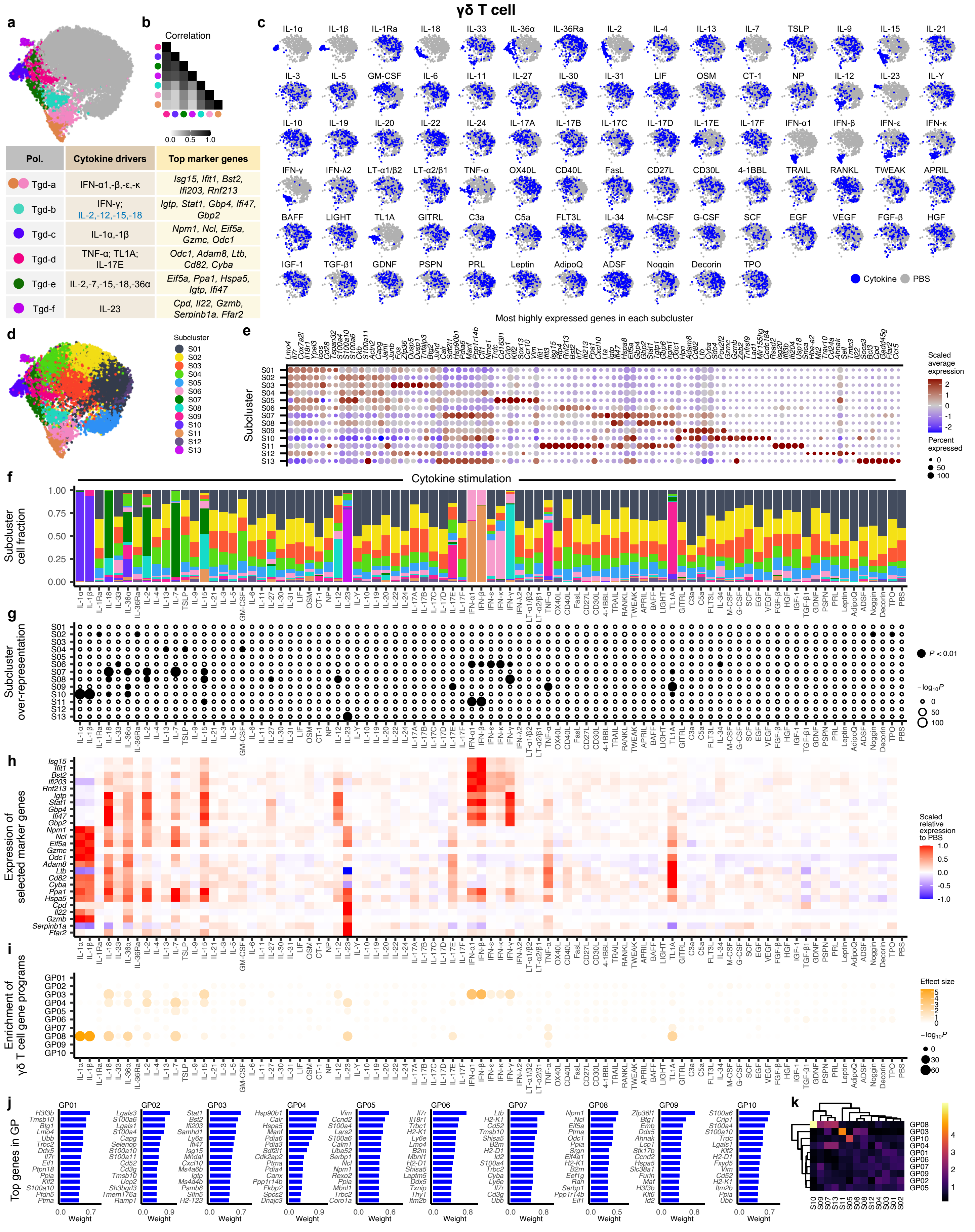

Pol.	Cytokine drivers	Top marker genes
Tgd-a	IFN-α1,-β,-ε,-κ	*Isg15, Ifit1, Bst2, Ifi203, Rnf213*
Tgd-b	IFN-γ; IL-2,-12,-15,-18	*Igtp, Stat1, Gbp4, Ifi47, Gbp2*
Tgd-c	IL-1α,-1β	*Npm1, Ncl, Eif5a, Gzmc, Odc1*
Tgd-d	TNF-α; TL1A; IL-17E	*Odc1, Adam8, Ltb, Cd82, Cyba*
Tgd-e	IL-2,-7,-15,-18,-36α	*Eif5a, Ppa1, Hspa5, Igtp, Ifi47*
Tgd-f	IL-23	*Cpd, Il22, Gzmb, Serpinb1a, Ffar2*

Supplementary Fig. 4 | γδ T cell responses to cytokines: polarization states, subclusters, marker gene expression, and gene programs. See next page for caption.

Supplementary Fig. 4 | γδ T cell responses to cytokines: polarization states, subclusters, marker gene expression, and gene programs.

a, Top, UMAP visualization of γδ T cells for all cytokines, colored by polarization states; bottom, table with cell type polarization states (left column), single cytokine drivers (middle column), and top marker genes (right column); reproduced from **Fig. 3** for ease of reference. **b**, Pairwise Pearson correlation coefficients between polarization states. **c**, UMAP visualization of γδ T cells shown independently for each cytokine treatment, colored by cytokine treatment (blue) or PBS treatment control (gray). **d**, UMAP visualization of γδ T cells for all cytokine or PBS treatment; cells colored for γδ T cell Louvain subclusters. **e**, Top overexpressed genes in each Louvain subcluster in **d**; color, column-scaled average expression; size of circle, percentage of cells in the subcluster expressing each gene. **f-i**, γδ T cell responses to each cytokine stimulation. **f**, Fraction of cells per subcluster in each cytokine treatment. Colors represent subclusters defined in **d**. **g**, Enrichment of each subcluster in each cytokine treatment; size of circle, Bonferroni-adjusted *P*-value of hypergeometric test relative to PBS; black fills, $P < 0.01$. **h**, Row-normalized relative expression of representative marker genes of each polarization state in cytokine-treated vs. PBS-treated cells. **i**, Enrichment of γδ T cell gene programs obtained from NMF analysis of all γδ T cell in cytokine-treated cells relative to PBS-treated cells; size of circle, FDR-adjusted *P*-value from two-sided Wilcoxon rank-sum test; shade, effect size representing the mean difference in gene program weight. **j**, Top weighted genes in each gene program in **i**. **k**, Average gene program weight in each subcluster. Rows and columns were hierarchically clustered using the complete-linkage method on Euclidean distances.

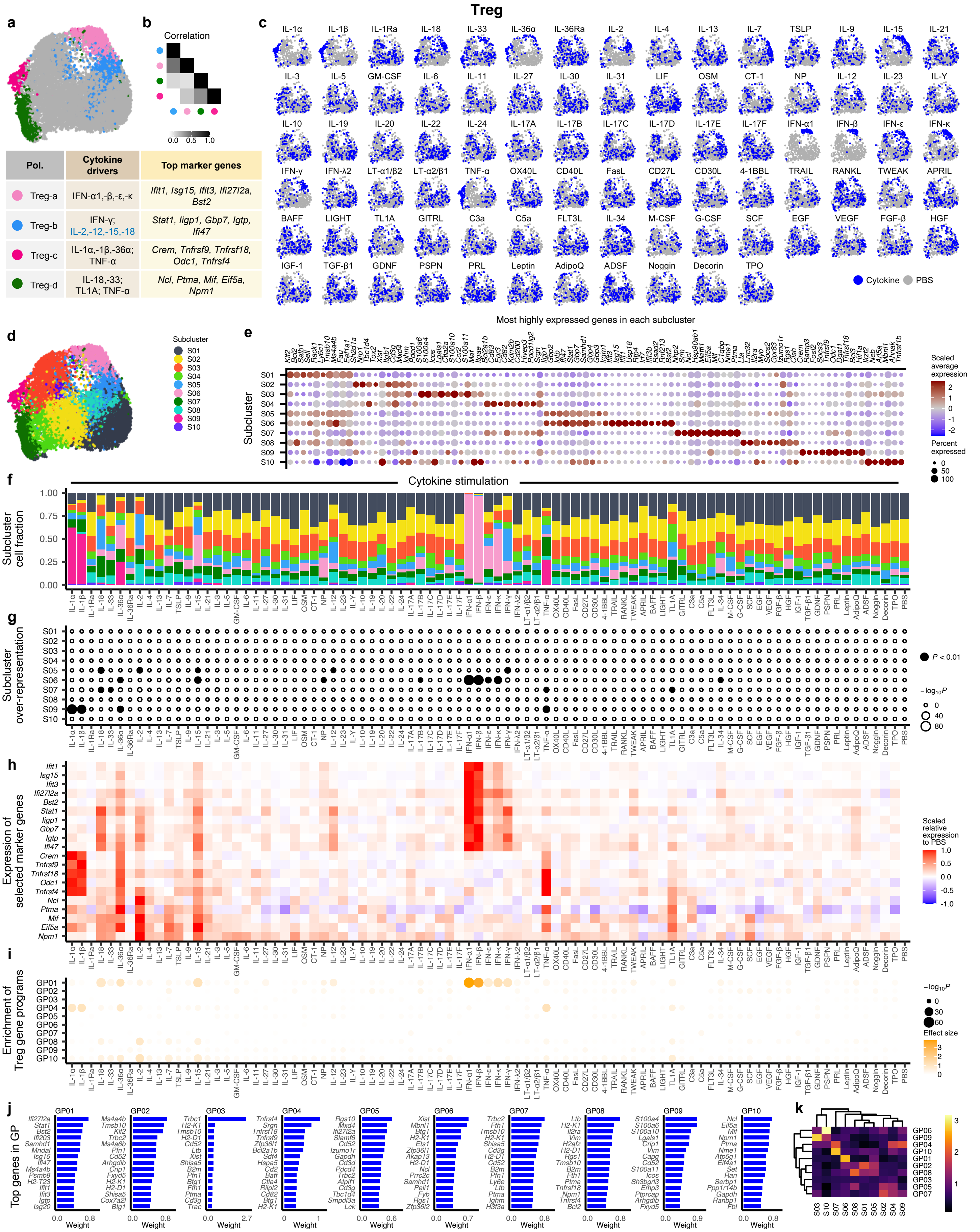

Supplementary Fig. 5 | Treg responses to cytokines: polarization states, subclusters, marker gene expression, and gene programs.
See next page for caption.

Supplementary Fig. 5 | Treg responses to cytokines: polarization states, subclusters, marker gene expression, and gene programs.

a, Top, UMAP visualization of Tregs for all cytokines, colored by polarization states; bottom, table with cell type polarization states (left column), single cytokine drivers (middle column), and top marker genes (right column); reproduced from **Fig. 3** for ease of reference. **b**, Pairwise Pearson correlation coefficients between polarization states. **c**, UMAP visualization of Tregs shown independently for each cytokine treatment, colored by cytokine treatment (blue) or PBS treatment control (gray). **d**, UMAP visualization of Tregs for all cytokine or PBS treatment; cells colored for Treg Louvain subclusters. **e**, Top overexpressed genes in each Louvain subcluster in **d**; color, column-scaled average expression; size of circle, percentage of cells in the subcluster expressing each gene. **f-i**, Treg responses to each cytokine stimulation. **f**, Fraction of cells per subcluster in each cytokine treatment. Colors represent subclusters defined in **d**. **g**, Enrichment of each subcluster in each cytokine treatment; size of circle, Bonferroni-adjusted *P*-value of hypergeometric test relative to PBS; black fills, $P < 0.01$. **h**, Row-normalized relative expression of representative marker genes of each polarization state in cytokine-treated vs. PBS-treated cells. **i**, Enrichment of Treg gene programs obtained from NMF analysis of all Tregs in cytokine-treated cells relative to PBS-treated cells; size of circle, FDR-adjusted *P*-value from two-sided Wilcoxon rank-sum test; shade, effect size representing the mean difference in gene program weight. **j**, Top weighted genes in each gene program in **i**. **k**, Average gene program weight in each subcluster. Rows and columns were hierarchically clustered using the complete-linkage method on Euclidean distances.

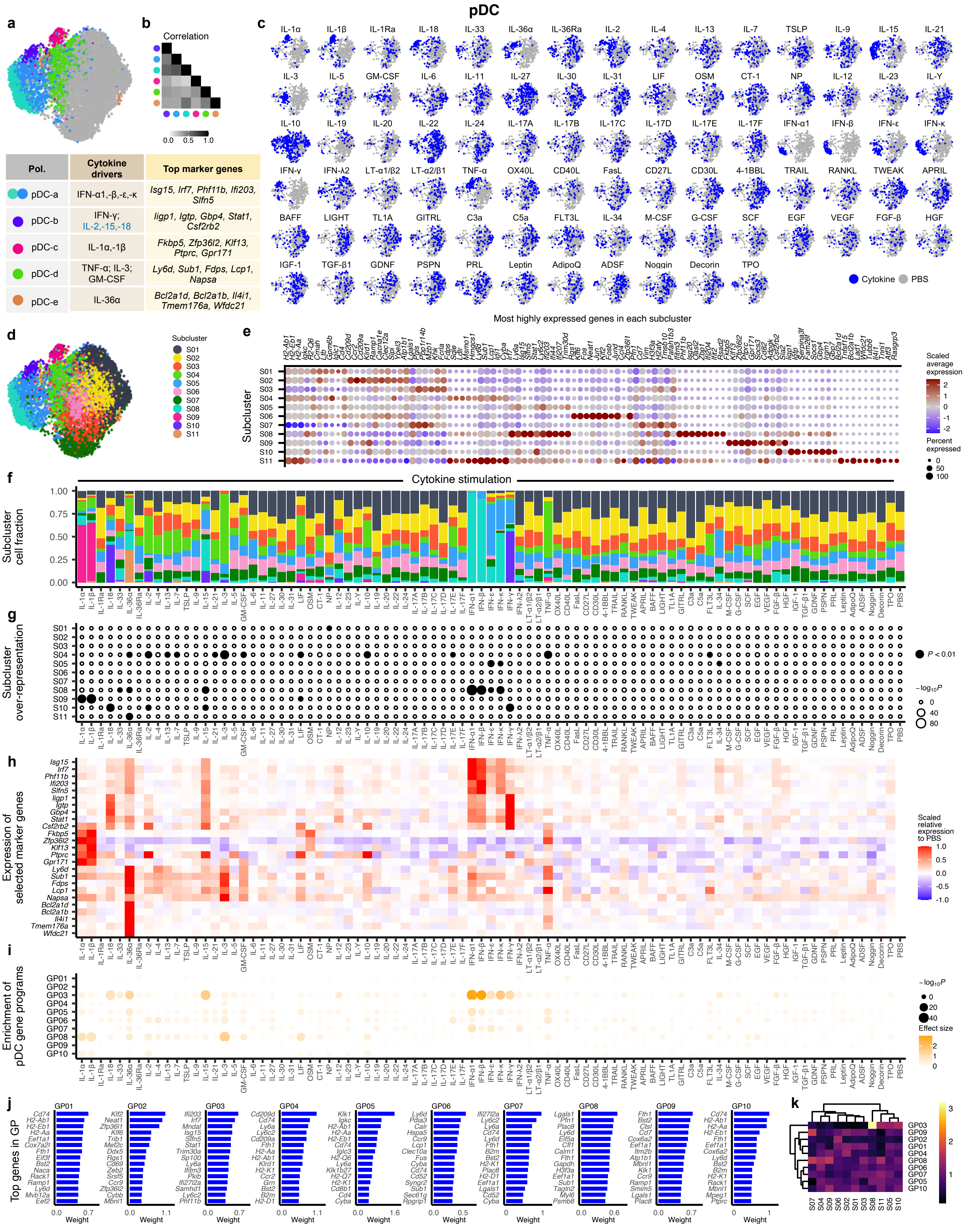

Supplementary Fig. 6 | pDC responses to cytokines: polarization states, subclusters, marker gene expression, and gene programs.
See next page for caption.

Supplementary Fig. 6 | pDC responses to cytokines: polarization states, subclusters, marker gene expression, and gene programs.

a, Top, UMAP visualization of pDCs for all cytokines, colored by polarization states; bottom, table with cell type polarization states (left column), single cytokine drivers (middle column), and top marker genes (right column); reproduced from **Fig. 3** for ease of reference. **b**, Pairwise Pearson correlation coefficients between polarization states. **c**, UMAP visualization of pDCs shown independently for each cytokine treatment, colored by cytokine treatment (blue) or PBS treatment control (gray). **d**, UMAP visualization of pDCs for all cytokine or PBS treatment; cells colored for pDC Louvain subclusters. **e**, Top overexpressed genes in each Louvain subcluster in **d**; color, column-scaled average expression; size of circle, percentage of cells in the subcluster expressing each gene. **f-i**, pDC responses to each cytokine stimulation. **f**, Fraction of cells per subcluster in each cytokine treatment. Colors represent subclusters defined in **d**. **g**, Enrichment of each subcluster in each cytokine treatment; size of circle, Bonferroni-adjusted *P*-value of hypergeometric test relative to PBS; black fills, $P < 0.01$. **h**, Row-normalized relative expression of representative marker genes of each polarization state in cytokine-treated vs. PBS-treated cells. **i**, Enrichment of pDC gene programs obtained from NMF analysis of all pDCs in cytokine-treated cells relative to PBS-treated cells; size of circle, FDR-adjusted *P*-value from two-sided Wilcoxon rank-sum test; shade, effect size representing the mean difference in gene program weight. **j**, Top weighted genes in each gene program in **i**. **k**, Average gene program weight in each subcluster. Rows and columns were hierarchically clustered using the complete-linkage method on Euclidean distances.

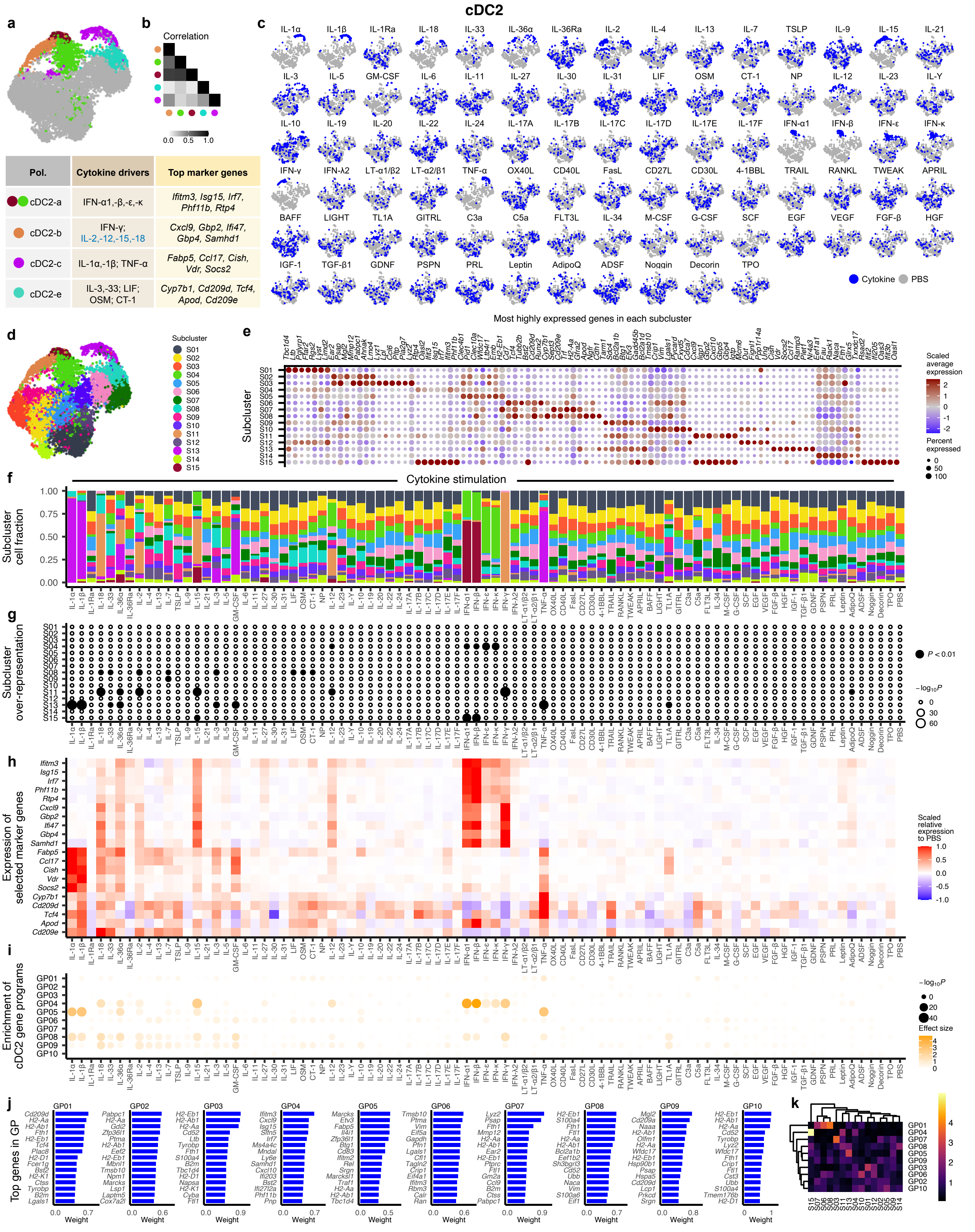

Supplementary Fig. 7 | cDC2 responses to cytokines: polarization states, subclusters, marker gene expression, and gene programs. See next page for caption.

Supplementary Fig. 7 | cDC2 responses to cytokines: polarization states, subclusters, marker gene expression, and gene programs.

a, Top, UMAP visualization of cDC2 cells for all cytokines, colored by polarization states; bottom, table with cell type polarization states (left column), single cytokine drivers (middle column), and top marker genes (right column); reproduced from **Fig. 3** for ease of reference. **b**, Pairwise Pearson correlation coefficients between polarization states. **c**, UMAP visualization of cDC2 cells shown independently for each cytokine treatment, colored by cytokine treatment (blue) or PBS treatment control (gray). **d**, UMAP visualization of cDC2 cells for all cytokine or PBS treatment; cells colored for cDC2 Louvain subclusters. **e**, Top overexpressed genes in each Louvain subcluster in **d**; color, column-scaled average expression; size of circle, percentage of cells in the subcluster expressing each gene. **f-i**, cDC2 responses to each cytokine stimulation. **f**, Fraction of cells per subcluster in each cytokine treatment. Colors represent subclusters defined in **d**. **g**, Enrichment of each subcluster in each cytokine treatment; size of circle, Bonferroni-adjusted *P*-value of hypergeometric test relative to PBS; black fills, $P < 0.01$. **h**, Row-normalized relative expression of representative marker genes of each polarization state in cytokine-treated vs. PBS-treated cells. **i**, Enrichment of cDC2 gene programs obtained from NMF analysis of all cDC2 cells in cytokine-treated cells relative to PBS-treated cells; size of circle, FDR-adjusted *P*-value from two-sided Wilcoxon rank-sum test; shade, effect size representing the mean difference in gene program weight. **j**, Top weighted genes in each gene program in **i**. **k**, Average gene program weight in each subcluster. Rows and columns were hierarchically clustered using the complete-linkage method on Euclidean distances.

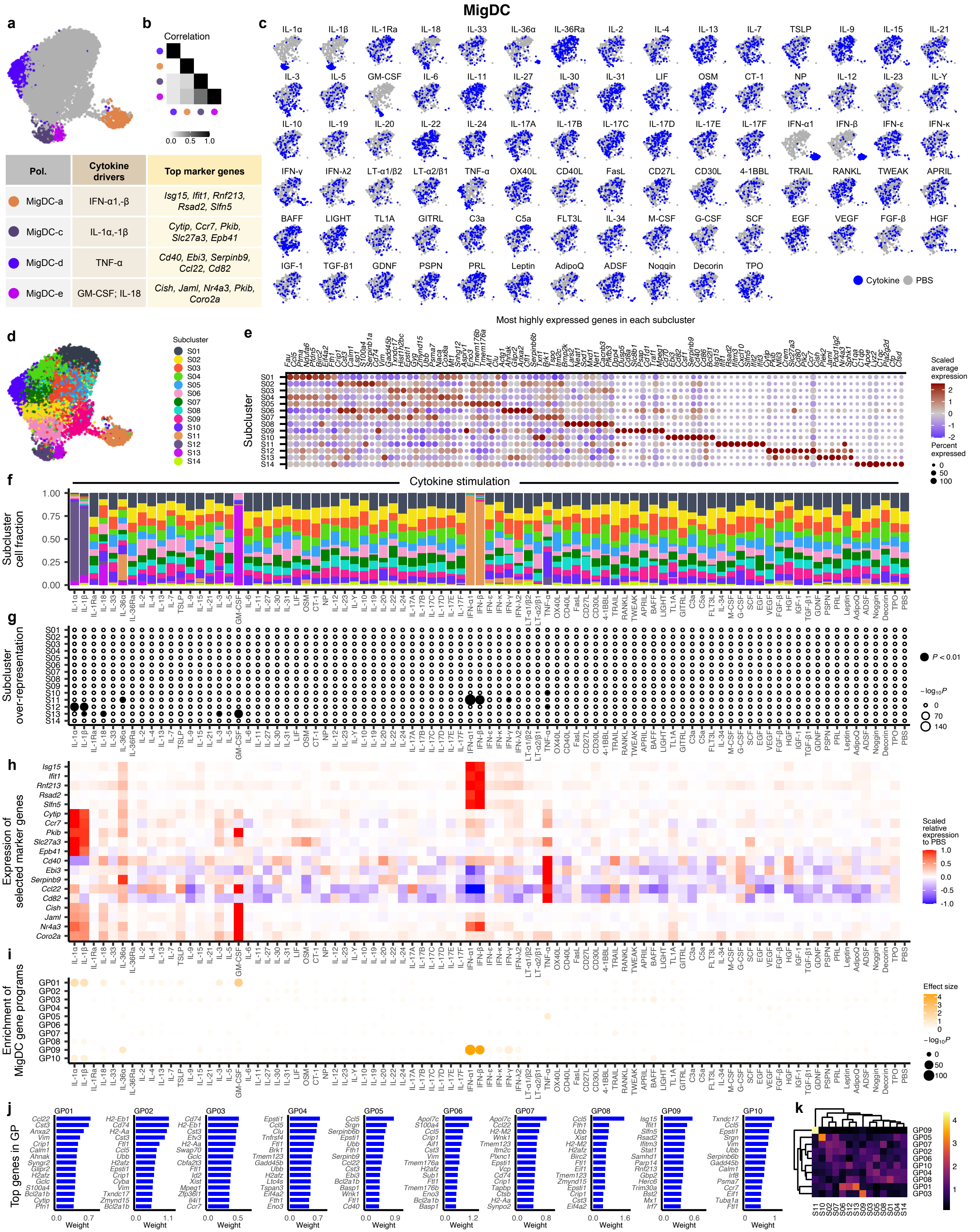

Supplementary Fig. 8 | MigDC responses to cytokines: polarization states, subclusters, marker gene expression, and gene programs. See next page for caption.

Supplementary Fig. 8 | MigDC responses to cytokines: polarization states, subclusters, marker gene expression, and gene programs.

a, Top, UMAP visualization of MigDCs for all cytokines, colored by polarization states; bottom, table with cell type polarization states (left column), single cytokine drivers (middle column), and top marker genes (right column); reproduced from **Fig. 3** for ease of reference. **b**, Pairwise Pearson correlation coefficients between polarization states. **c**, UMAP visualization of MigDCs shown independently for each cytokine treatment, colored by cytokine treatment (blue) or PBS treatment control (gray). **d**, UMAP visualization of MigDCs for all cytokine or PBS treatment; cells colored for MigDC Louvain subclusters. **e**, Top overexpressed genes in each Louvain subcluster in **d**; color, column-scaled average expression; size of circle, percentage of cells in the subcluster expressing each gene. **f-i**, MigDC responses to each cytokine stimulation. **f**, Fraction of cells per subcluster in each cytokine treatment. Colors represent subclusters defined in **d**. **g**, Enrichment of each subcluster in each cytokine treatment; size of circle, Bonferroni-adjusted *P*-value of hypergeometric test relative to PBS; black fills, $P < 0.01$. **h**, Row-normalized relative expression of representative marker genes of each polarization state in cytokine-treated vs. PBS-treated cells. **i**, Enrichment of MigDC gene programs obtained from NMF analysis of all MigDCs in cytokine-treated cells relative to PBS-treated cells; size of circle, FDR-adjusted *P*-value from two-sided Wilcoxon rank-sum test; shade, effect size representing the mean difference in gene program weight. **j**, Top weighted genes in each gene program in **i**. **k**, Average gene program weight in each subcluster. Rows and columns were hierarchically clustered using the complete-linkage method on Euclidean distances.

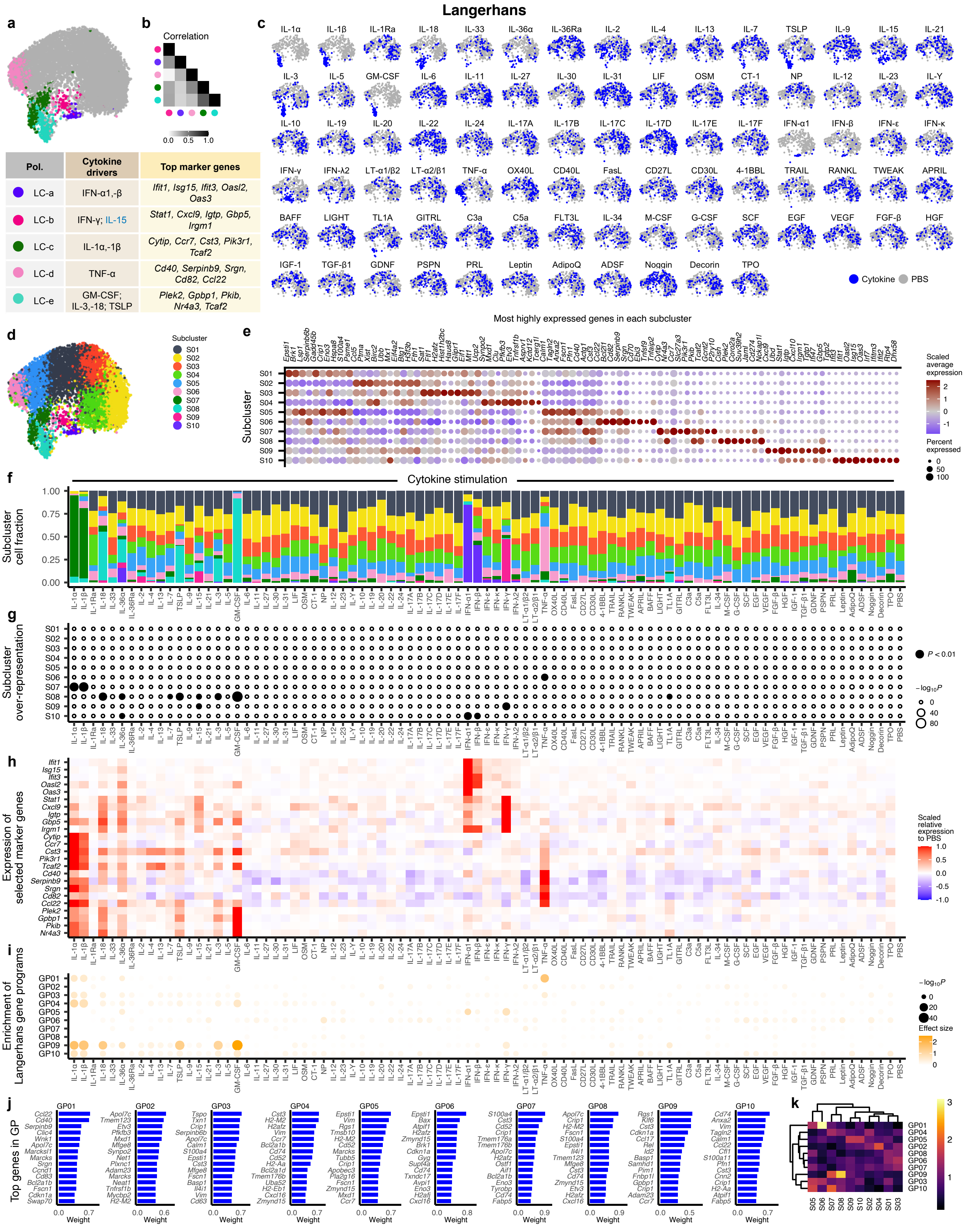

Supplementary Fig. 9 | Langerhans cell responses to cytokines: polarization states, subclusters, marker gene expression, and gene programs. See next page for caption.

Supplementary Fig. 9 | Langerhans cell responses to cytokines: polarization states, subclusters, marker gene expression, and gene programs.

a, Top, UMAP visualization of Langerhans cells for all cytokines, colored by polarization states; bottom, table with cell type polarization states (left column), single cytokine drivers (middle column), and top marker genes (right column); reproduced from **Fig. 3** for ease of reference. **b**, Pairwise Pearson correlation coefficients between polarization states. **c**, UMAP visualization of Langerhans cells shown independently for each cytokine treatment, colored by cytokine treatment (blue) or PBS treatment control (gray). **d**, UMAP visualization of Langerhans cells for all cytokine or PBS treatment; cells colored for Langerhans cell Louvain subclusters. **e**, Top overexpressed genes in each Louvain subcluster in **d**; color, column-scaled average expression; size of circle, percentage of cells in the subcluster expressing each gene. **f-i**, Langerhans cell responses to each cytokine stimulation. **f**, Fraction of cells per subcluster in each cytokine treatment. Colors represent subclusters defined in **d**. **g**, Enrichment of each subcluster in each cytokine treatment; size of circle, Bonferroni-adjusted *P*-value of hypergeometric test relative to PBS; black fills, $P < 0.01$. **h**, Row-normalized relative expression of representative marker genes of each polarization state in cytokine-treated vs. PBS-treated cells. **i**, Enrichment of Langerhans cell gene programs obtained from NMF analysis of all Langerhans cells in cytokine-treated cells relative to PBS-treated cells; size of circle, FDR-adjusted *P*-value from two-sided Wilcoxon rank-sum test; shade, effect size representing the mean difference in gene program weight. **j**, Top weighted genes in each gene program in **i**. **k**, Average gene program weight in each subcluster. Rows and columns were hierarchically clustered using the complete-linkage method on Euclidean distances.

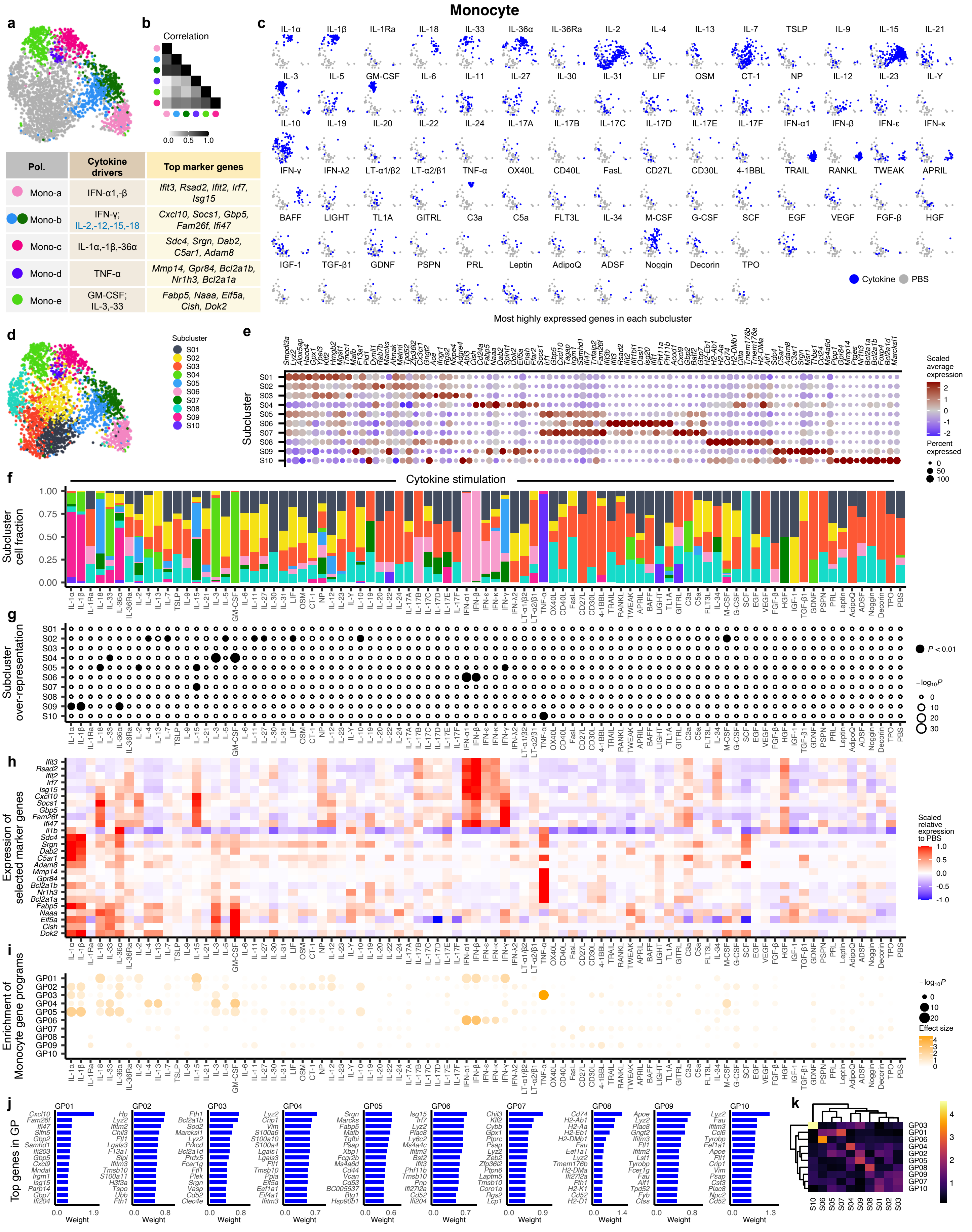

Supplementary Fig. 10 | Monocyte responses to cytokines: polarization states, subclusters, marker gene expression, and gene programs. See next page for caption.

Supplementary Fig. 10 | Monocyte responses to cytokines: polarization states, subclusters, marker gene expression, and gene programs.

a, Top, UMAP visualization of monocytes for all cytokines, colored by polarization states; bottom, table with cell type polarization states (left column), single cytokine drivers (middle column), and top marker genes (right column); reproduced from **Fig. 3** for ease of reference. **b**, Pairwise Pearson correlation coefficients between polarization states. **c**, UMAP visualization of monocytes shown independently for each cytokine treatment, colored by cytokine treatment (blue) or PBS treatment control (gray). **d**, UMAP visualization of monocytes for all cytokine or PBS treatment; cells colored for monocyte Louvain subclusters. **e**, Top overexpressed genes in each Louvain subcluster in **d**; color, column-scaled average expression; size of circle, percentage of cells in the subcluster expressing each gene. **f-i**, Monocyte responses to each cytokine stimulation. **f**, Fraction of cells per subcluster in each cytokine treatment. Colors represent subclusters defined in **d**. **g**, Enrichment of each subcluster in each cytokine treatment; size of circle, Bonferroni-adjusted P-value of hypergeometric test relative to PBS; black fills, $P < 0.01$. **h**, Row-normalized relative expression of representative marker genes of each polarization state in cytokine-treated vs. PBS-treated cells. **i**, Enrichment of monocyte gene programs obtained from NMF analysis of all monocytes in cytokine-treated cells relative to PBS-treated cells; size of circle, FDR-adjusted P-value from two-sided Wilcoxon rank-sum test; shade, effect size representing the mean difference in gene program weight. **j**, Top weighted genes in each gene program in **i**. **k**, Average gene program weight in each subcluster. Rows and columns were hierarchically clustered using the complete-linkage method on Euclidean distances.

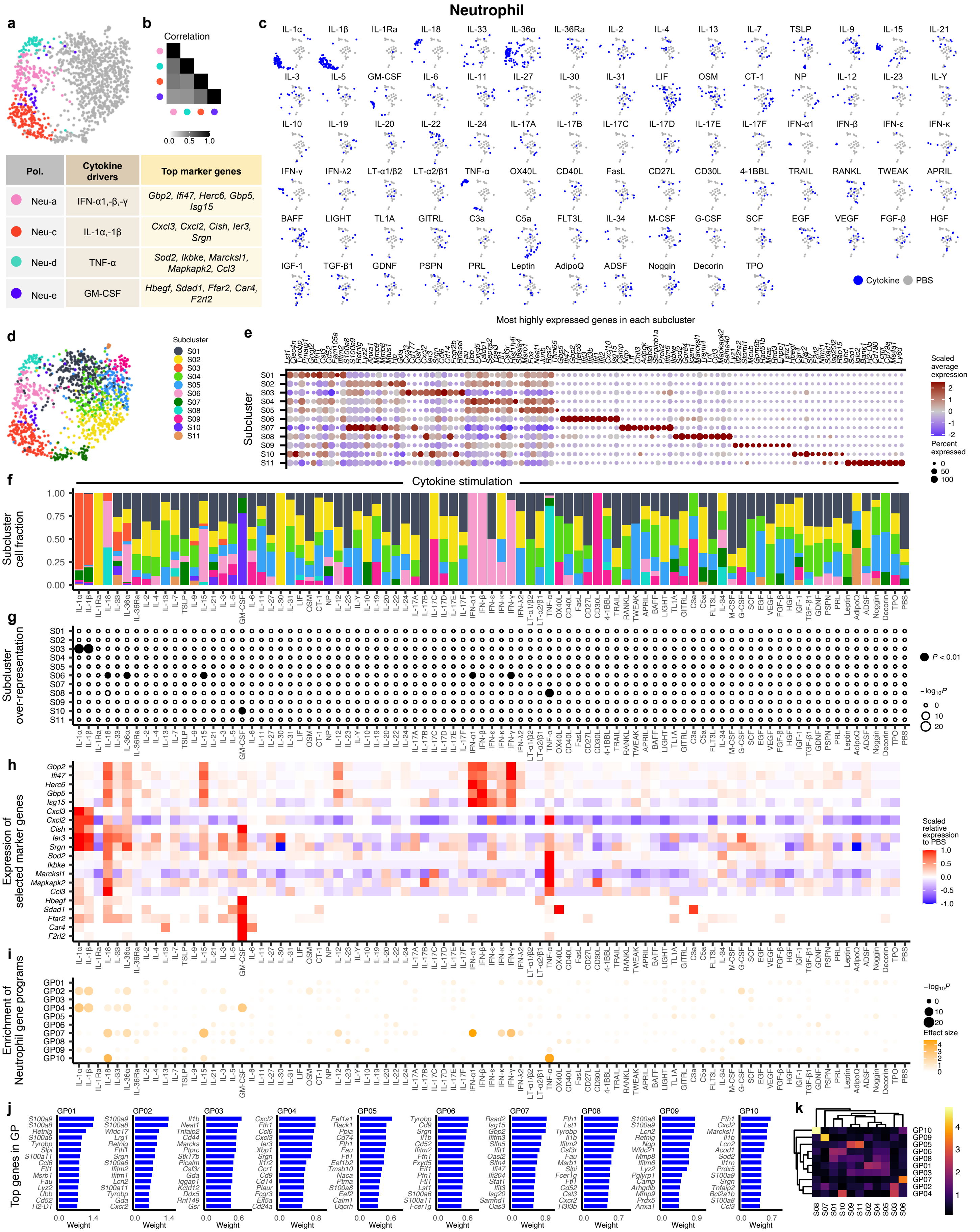

Supplementary Fig. 11 | Neutrophil responses to cytokines: polarization states, subclusters, marker gene expression, and gene programs. See next page for caption.

Supplementary Fig. 11 | Neutrophil responses to cytokines: polarization states, subclusters, marker gene expression, and gene programs.

a, Top, UMAP visualization of neutrophils for all cytokines, colored by polarization states; bottom, table with cell type polarization states (left column), single cytokine drivers (middle column), and top marker genes (right column); reproduced from **Fig. 3** for ease of reference. **b**, Pairwise Pearson correlation coefficients between polarization states. **c**, UMAP visualization of neutrophils shown independently for each cytokine treatment, colored by cytokine treatment (blue) or PBS treatment control (gray). **d**, UMAP visualization of neutrophils for all cytokine or PBS treatment; cells colored for neutrophil Louvain subclusters. **e**, Top overexpressed genes in each Louvain subcluster in **d**; color, column-scaled average expression; size of circle, percentage of cells in the subcluster expressing each gene. **f-i**, Neutrophil responses to each cytokine stimulation. **f**, Fraction of cells per subcluster in each cytokine treatment. Colors represent subclusters defined in **d**. **g**, Enrichment of each subcluster in each cytokine treatment; size of circle, Bonferroni-adjusted *P*-value of hypergeometric test relative to PBS; black fills, $P < 0.01$. **h**, Row-normalized relative expression of representative marker genes of each polarization state in cytokine-treated vs. PBS-treated cells. **i**, Enrichment of neutrophil gene programs obtained from NMF analysis of all neutrophils in cytokine-treated cells relative to PBS-treated cells; size of circle, FDR-adjusted *P*-value from two-sided Wilcoxon rank-sum test; shade, effect size representing the mean difference in gene program weight. **j**, Top weighted genes in each gene program in **i**. **k**, Average gene program weight in each subcluster. Rows and columns were hierarchically clustered using the complete-linkage method on Euclidean distances.

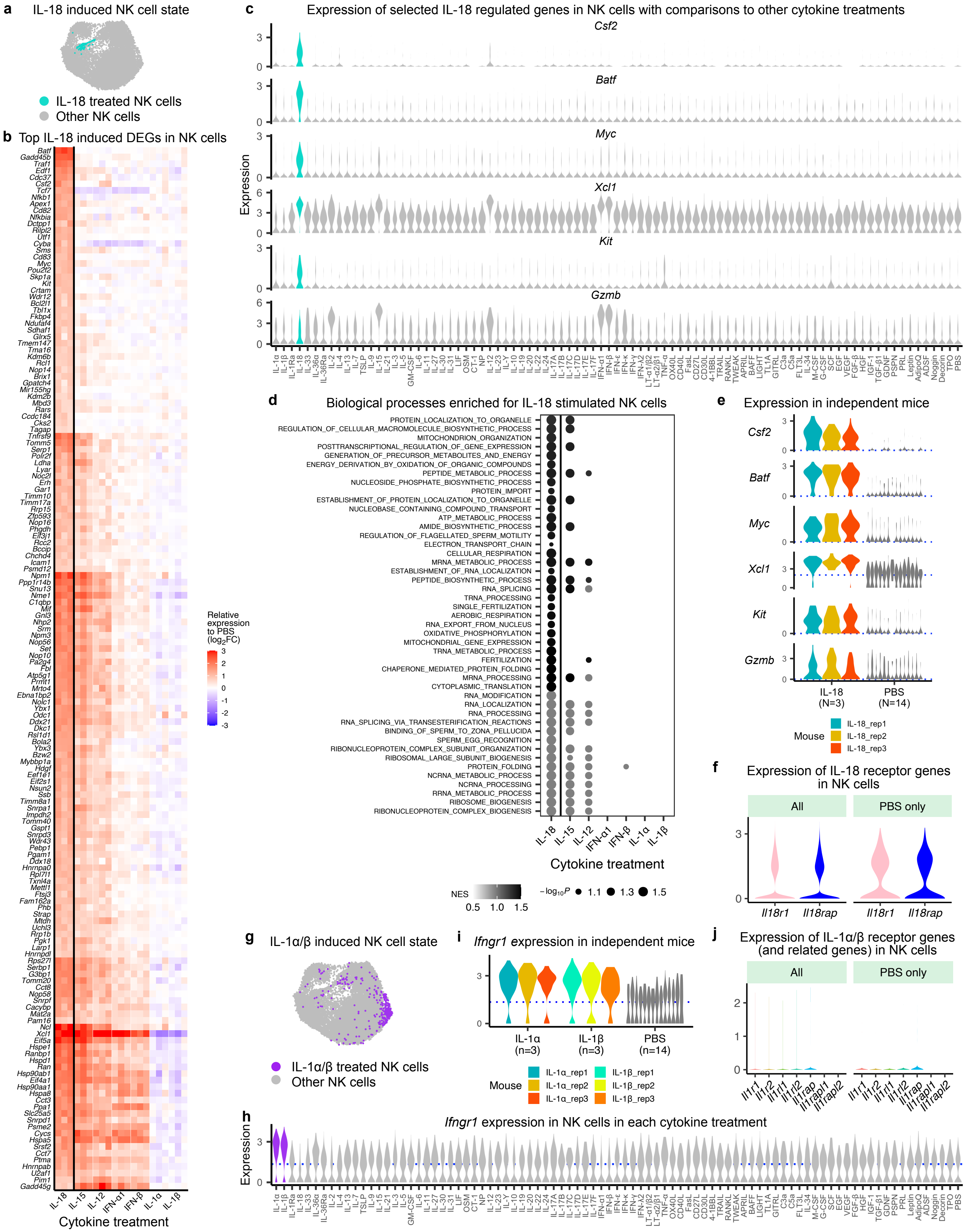

Supplementary Fig. 12 | Further characterization of IL-18 induced NK-f polarization state and IL-1α/β induced NK-c polarization state in NK cells.

See next page for caption.

Supplementary Fig. 12 | Further characterization of IL-18 induced NK-f polarization state and IL-1α/β induced NK-c polarization state in NK cells.

a-f, NK-f polarization state induced by IL-18. **a,** UMAP visualization of NK cells for all cytokine or PBS treatment, colored by IL-18 treatment. Reproduced from **Extended Data Fig. 6c** IL-18 subpanel. **b**, Top IL-18 regulated genes in NK cells ($log_2FC > 1.1$ and FDR-adjusted $P < 0.01$, two-sided Wilcoxon rank-sum test), colored by mean log_2FC in expression relative to PBS treatment. Major cytokines inducing other NK cell polarization states are shown for comparison for IL-18-regulated genes. 3 independent mice for each cytokine treatment are shown in adjacent columns. **c**, Violin plot showing expression of selected IL-18 regulated genes in NK cells in response to each of the 86 cytokines. IL-18 treated cells are colored in cyan. **d**, Biological processes enriched in NK cells stimulated with IL-18 compared to PBS. Other cytokines shown as a comparison as in **b**. Shade represents normalized enrichment score (NES); size represents FDR-adjusted *P*-value. **e**, Violin plot showing expression of IL-18 regulated genes in 3 independent mice treated with IL-18 or in 14 independent mice treated with PBS; dotted line, median expression in PBS-treated cells. **f**, Violin plot showing expression of genes encoding IL-18 receptors in NK cells, for all cells treated with any cytokine or with PBS only. **g-j,** NK-c polarization state induced by IL-1α/β. **g**, UMAP visualization of NK cells for all cytokine or PBS treatment, colored by IL-1α/β treatment. **h**, Violin plot showing expression of IL-1α/β-regulated *Ifngr1* expression in NK cells in each cytokine treatment; dotted line, median expression of PBS-treated cells; reproduced from **Fig. 3p** *Ifngr1* panel for ease of reference. **i**, Violin plot showing expression of *Ifngr1* in 6 independent mice treated with IL-1α/β or in 14 independent mice treated with PBS; dotted line, median expression of PBS-treated cells. **j**, Violin plot showing expression of genes encoding IL-1α/β receptors and related receptors in NK cells; shown for all cells treated with any cytokine or with PBS only.

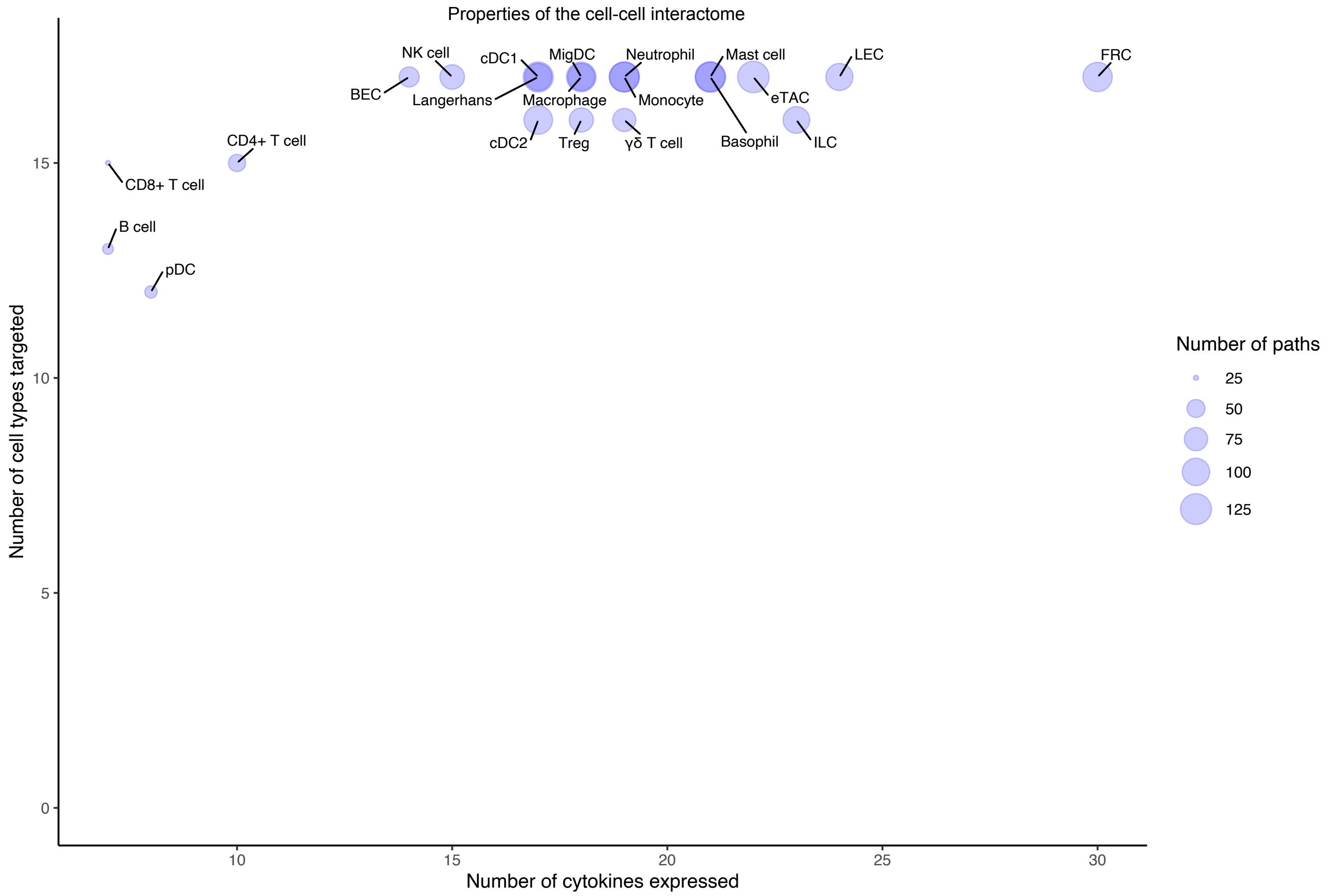

Supplementary Fig. 13 | Cell type properties in the cell-cell interactome.

Properties of Extended Data Fig. 11. For each cell type (a circle), showing the number of cytokines expressed and the total number of cell types targeted through any cytokine. Size of the circle is proportional to the number of paths in the cell-cell interactome.

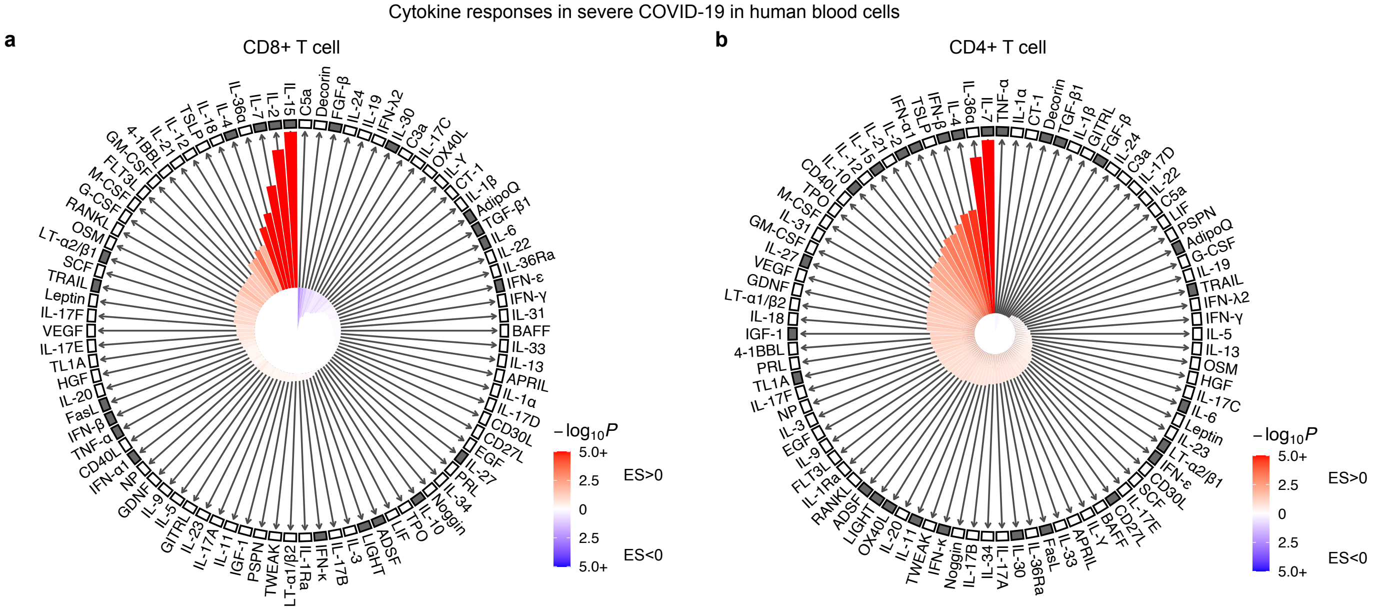

Supplementary Fig. 14 | Additional IREA software output on human COVID-19 blood samples.

a-b. IREA analysis on peripheral blood cells collected from severe COVID-19 patients relative to healthy volunteers. IREA compass plots showing enrichment scores for each of 86 cytokines in CD8+ T cells and CD4+ T cells in ventilated COVID-19 patients relative to healthy controls. Bar length represents enrichment score, shade represents FDR adjusted *P*-value (two-sided Wilcoxon rank-sum test), with darker colors representing more significant enrichment (red: enriched in ventilated COVID-19 patients, blue: enriched in healthy control). Cytokines with receptors expressed are indicated by black filled boxes.
