# Supplementary material for: Dictionary of immune responses to cytokines at single-cell resolution
Source: Nature. 2023 Dec 6;625(7994):377–84. doi: 10.1038/s41586-023-06816-9 (PMC10781646; doi:10.1038/s41586-023-06816-9)
Supplement: Supplementary file 2 — Reporting Summary [file 41586_2023_6816_MOESM2_ESM.pdf]

nature portfolio

Corresponding author(s): Ang Cui, Nir Hacohen

Last updated by author(s): Aug 30, 2022

# Reporting Summary

Nature Portfolio wishes to improve the reproducibility of the work that we publish. This form provides structure for consistency and transparency in reporting. For further information on Nature Portfolio policies, see our Editorial Policies and the Editorial Policy Checklist.

## Statistics

For all statistical analyses, confirm that the following items are present in the figure legend, table legend, main text, or Methods section.

| n/a | Confirmed | |
|---|---|---|
| ☐ | ☒ | The exact sample size (*n*) for each experimental group/condition, given as a discrete number and unit of measurement |
| ☐ | ☒ | A statement on whether measurements were taken from distinct samples or whether the same sample was measured repeatedly |
| ☐ | ☒ | The statistical test(s) used AND whether they are one- or two-sided<br>*Only common tests should be described solely by name; describe more complex techniques in the Methods section.* |
| ☐ | ☒ | A description of all covariates tested |
| ☐ | ☒ | A description of any assumptions or corrections, such as tests of normality and adjustment for multiple comparisons |
| ☐ | ☒ | A full description of the statistical parameters including central tendency (e.g. means) or other basic estimates (e.g. regression coefficient) AND variation (e.g. standard deviation) or associated estimates of uncertainty (e.g. confidence intervals) |
| ☐ | ☒ | For null hypothesis testing, the test statistic (e.g. *F*, *t*, *r*) with confidence intervals, effect sizes, degrees of freedom and *P* value noted<br>*Give P values as exact values whenever suitable.* |
| ☒ | ☐ | For Bayesian analysis, information on the choice of priors and Markov chain Monte Carlo settings |
| ☒ | ☐ | For hierarchical and complex designs, identification of the appropriate level for tests and full reporting of outcomes |
| ☐ | ☒ | Estimates of effect sizes (e.g. Cohen's *d*, Pearson's *r*), indicating how they were calculated |

*Our web collection on statistics for biologists contains articles on many of the points above.*

## Software and code

Policy information about availability of computer code

| | |
|---|---|
| Data collection | No software was used for data collection. |
| Data analysis | The raw bcl sequencing data was processed using CellRanger v3.0. Hashtag library FASTQ files were processed through the CITE-seq-Count tool (v1.4.3, https://github.com/Hoohm/CITE-seq-Count). The Seurat R package (v4.1) was used to analyze single-cell RNA-sequencing data. The R packages NMFN (v2.0) and clusterProfiler (v4.2.1) were used. Analyses were performed in R (v4.0). |

For manuscripts utilizing custom algorithms or software that are central to the research but not yet described in published literature, software must be made available to editors and reviewers. We strongly encourage code deposition in a community repository (e.g. GitHub). See the Nature Portfolio guidelines for submitting code & software for further information.

## Data

Policy information about availability of data

All manuscripts must include a data availability statement. This statement should provide the following information, where applicable:

- Accession codes, unique identifiers, or web links for publicly available datasets
- A description of any restrictions on data availability
- For clinical datasets or third party data, please ensure that the statement adheres to our policy

The Immune Dictionary interactive web portal can be accessed at www.immune-dictionary.org, where the single-cell transcriptomic data generated in this study are made publicly available through a user-friendly interface. Raw fastq files of the data are available on Gene Expression Omnibus (GEO) under the accession number GSE202186.
In addition, the following publicly available datasets were used. The MSigDB database was used for annotating biological processes and can be accessed at https://

www.gsea-msigdb.org/gsea/msigdb. Mouse and human homolog gene conversion was based on the NCBI HomoloGene database (release 68). The mouse tumor dataset is available on GEO under the accession number GSE119352. The human COVID-19 dataset is available on https://www.covid19cellatlas.org.

# Field-specific reporting

Please select the one below that is the best fit for your research. If you are not sure, read the appropriate sections before making your selection.

☒ Life sciences ☐ Behavioural & social sciences ☐ Ecological, evolutionary & environmental sciences

For a reference copy of the document with all sections, see nature.com/documents/nr-reporting-summary-flat.pdf

# Life sciences study design

All studies must disclose on these points even when the disclosure is negative.

| | |
|---|---|
| Sample size | Transcriptomic profiles of 386,703 independent cells were measured from 272 mice. At least 3 independent mice were used for each condition to allow for standard statistical tests. |
| Data exclusions | Pre-established quality control criteria were used: we included cell barcodes with >500 genes, >1,000 UMIs, and <10% mitochondrial gene content. Multiplets (more than one cells mapped to the same barcode) that arise from the microfluidics technology were identified based on the Methods provided and were excluded from downstream analyses. |
| Replication | For each condition, at least 3 independent animals were used. The results were reproducible across animal replicates. |
| Randomization | Animals of the same sex, age group, and genetic background were randomly allocated to the experimental groups. The animal replicates of each condition were randomized into different experimental batches to ensure that batch effects, if any, do not influence biological interpretations. |
| Blinding | The investigators performing animal experiments and RNA-sequencing were blinded from each other during data collection. Single-cell analysis was performed computationally in a completely unbiased fashion. |

# Reporting for specific materials, systems and methods

We require information from authors about some types of materials, experimental systems and methods used in many studies. Here, indicate whether each material, system or method listed is relevant to your study. If you are not sure if a list item applies to your research, read the appropriate section before selecting a response.

## Materials & experimental systems

| n/a | Involved in the study | |
|---|---|---|
| ☐ | ☒ | Antibodies |
| ☒ | ☐ | Eukaryotic cell lines |
| ☒ | ☐ | Palaeontology and archaeology |
| ☐ | ☒ | Animals and other organisms |
| ☒ | ☐ | Human research participants |
| ☒ | ☐ | Clinical data |
| ☒ | ☐ | Dual use research of concern |

## Methods

| n/a | Involved in the study | |
|---|---|---|
| ☒ | ☐ | ChIP-seq |
| ☒ | ☐ | Flow cytometry |
| ☒ | ☐ | MRI-based neuroimaging |

## Antibodies

| | |
|---|---|
| Antibodies used | TotalSeq™ anti-mouse hashtag 1-7 antibodies (BioLegend A0301-A0307; clones: M1/42, 30-F11; catalogue numbers: 155801, 155803, 155805, 155807, 155809, 155811, 155813).<br>Biotin anti-mouse CD19 (BioLegend; clone: 6D5; catalogue number: 115504).<br>Biotin anti-mouse CD3 (BioLegend; clone: 17A2; catalogue number: 100244).<br>TruStain fcX™ anti-mouse CD16/32 (Biolegend; clone: 93; catalogue number: 101320). |
| Validation | TotalSeq™ antibodies were validated in Stoeckius, M., et al, Genome Biology (2018).<br>All antibodies used are commercially available and have been validated by the manufacturer and prior publications. Validation data are available on BioLegend's website. |

## Animals and other organisms

Policy information about studies involving animals; ARRIVE guidelines recommended for reporting animal research

| | |
|---|---|
| Laboratory animals | Wild type female C57BL6/J mice at 11-15 weeks were studied. Mice were maintained on a 12-hour light/dark cycle at room temperature (21°C ± 2°C) and 40% ± 10% humidity. |

| | |
|---|---|
| Wild animals | No wild animals were used in the study. |
| Field-collected samples | No field-collected samples were used in the study. |
| Ethics oversight | All experiments were reviewed and approved by the Broad Institute's Institutional Animal Care and Use Committee (IACUC). |

Note that full information on the approval of the study protocol must also be provided in the manuscript.
